# A Unifying Mechanism for Cancer Cell Death through Ion Channel Activation by HAMLET

**DOI:** 10.1371/journal.pone.0058578

**Published:** 2013-03-07

**Authors:** Petter Storm, Thomas Kjaer Klausen, Maria Trulsson, James Ho CS, Marion Dosnon, Tomas Westergren, Yinxia Chao, Anna Rydström, Henry Yang, Stine Falsig Pedersen, Catharina Svanborg

**Affiliations:** 1 Department of Microbiology, Immunology and Glycobiology, Institute of Laboratory Medicine, Lund University, Lund, Sweden; 2 Department of Biology, University of Copenhagen, Copenhagen, Denmark; 3 Singapore Immunology Network, A*STAR, Singapore; Enzo Life Sciences, Inc., United States of America

## Abstract

Ion channels and ion fluxes control many aspects of tissue homeostasis. During oncogenic transformation, critical ion channel functions may be perturbed but conserved tumor specific ion fluxes remain to be defined. Here we used the tumoricidal protein-lipid complex HAMLET as a probe to identify ion fluxes involved in tumor cell death. We show that HAMLET activates a non-selective cation current, which reached a magnitude of 2.74±0.88 nA within 1.43±0.13 min from HAMLET application. Rapid ion fluxes were essential for HAMLET-induced carcinoma cell death as inhibitors (amiloride, BaCl_2_), preventing the changes in free cellular Na^+^ and K^+^ concentrations also prevented essential steps accompanying carcinoma cell death, including changes in morphology, uptake, global transcription, and MAP kinase activation. Through global transcriptional analysis and phosphorylation arrays, a strong ion flux dependent p38 MAPK response was detected and inhibition of p38 signaling delayed HAMLET-induced death. Healthy, differentiated cells were resistant to HAMLET challenge, which was accompanied by innate immunity rather than p38-activation. The results suggest, for the first time, a unifying mechanism for the initiation of HAMLET’s broad and rapid lethal effect on tumor cells. These findings are particularly significant in view of HAMLET’s documented therapeutic efficacy in human studies and animal models. The results also suggest that HAMLET offers a two-tiered therapeutic approach, killing cancer cells while stimulating an innate immune response in surrounding healthy tissues.

## Introduction

Ion channels are a prerequisite for normal cell function. They are highly conserved through evolution, and are activated by a wide variety of signals including mechanical forces, voltage, pH, matrix interactions and growth factor receptor activity [1], [2], [3], [4], [5], [6]. As a result, ion channels facilitate cellular adaptation to different physical environments, by adjusting proliferation and apoptosis, organ development and homeostasis. Ion channel activation has been proposed to regulate the activity of essential cellular signaling cascades, including p38 mitogen-activated protein kinases (MAPKs), small guanine triphosphate hydrolases (GTPases), the phosphatidyl-inositol-3-kinase (PI3K)-Akt pathway, NFκB- and Ca^2+^-dependent signaling pathways [7], [8]. Recently, ion channel dysregulation has been proposed to also promote malignant transformation, tumorigenesis and metastasis, suggesting a central role of ion channels for cancer development. Indeed, a wide range of ion channels, including Ca^2+^, K^+^, Na^+^, and Cl^−^ channels, and non-selective cation channels, have been implicated in various aspects of cancer development (for reviews, see [1], [9], [10], [11], [12], [13], [14], [15]).

HAMLET (human alpha-lactalbumin made lethal to tumor cells) is the first member of a new family of tumoricidal molecules with remarkable properties. Formed from partially unfolded α-lactalbumin and with oleic acid as an integral constituent [16], [17], HAMLET was discovered by serendipity when studying the ability of human milk to prevent bacteria from binding to host cells. Early *in vitro* experiments showed that HAMLET displays broad anti-tumor activity with a high degree of tumor selectivity [16], [17]. Subsequent therapeutic studies in patients and animal models have confirmed HAMLET´s tumoricidal activity and relative selectivity for tumor tissue *in vivo*. HAMLET treatment delayed tumor progression and led to increased survival in a rat glioblastoma xenograft model without evidence of cell death in healthy brain tissue [18]. Topical HAMLET administration removed or reduced the size of skin papillomas, as shown using a placebo-controlled protocol with a two-year follow up [19]. Local instillation of HAMLET in patients with bladder cancer rapidly killed tumor cells without toxic effects on healthy tissues surrounding the tumor [20] and therapeutic efficacy of HAMLET was demonstrated in a murine bladder cancer model [21]. Recently, peroral HAMLET administration has shown therapeutic as well as prophylactic efficacy against colon cancer in APC min mice (GUT, in press).

The sensitivity to HAMLET at least in part reflects increased c-Myc oncogene expression and dysregulated glycolysis in tumor cells [22], but the specific membrane interactions that initiate the HAMLET-induced cell death process have not been defined. Cellular responses to HAMLET are quite rapid compared to cell death inducers like FAS ligand or TNF-α [23], implying a more immediate and general mechanism for HAMLET sensing at the cell membrane than via traditional extrinsic apoptosis induction. In early studies, we detected rapid Ca^2+^ fluxes after tumor cell exposure to HAMLET [17], suggesting that ion channels and/or transporters might be activated. Recently, dramatic changes in the structure of artificial, receptor-free membranes and plasma membrane vesicles from cancer cells have been observed after HAMLET challenge [24]. Rounded, defined vesicles changed morphology to amorphous shapes, reflecting the formation of long membrane distensions with increased fluidity. A similar response to HAMLET was observed in plasma membrane vesicles from carcinoma cells, suggesting that direct membrane effects might contribute to the tumoricidal effect of HAMLET. Normal differentiated cells showed no evidence of membrane perturbation [24], suggesting that the membrane perturbations might characterize HAMLET sensitive tumor cells.

This study characterized ion fluxes triggered by HAMLET and examined their role in tumor cell death. We show that HAMLET activates a whole cell non-selective cation current, which we characterize electrophysiologically. Importantly, we show that ion fluxes are essential to initiate HAMLET-induced tumor cell death and to distinguish tumor cells from normal cells in this context. The effects of HAMLET on cancer cell viability were reversed by amiloride or BaCl_2_, non-specific inhibitors of several ion channels and transporters. In parallel, changes in morphology, ion fluxes, global transcription, MAPK signaling and p38 MAPK-dependent tumor cell death were also abrogated. Furthermore, the response of normal, differentiated cells to HAMLET showed marked differences from that of cancer cells, defined by the pattern of changes in global transcription, signaling pathway activation and survival. The establishment of a non-selective, amiloride-sensitive cation channel as essential to HAMLET-induced cancer cell death describes a hitherto unresolved mechanism and may represent a new therapeutic option.

## Materials and Methods

### HAMLET Production

α-Lactalbumin was purified from defatted human milk by ammonium sulfate precipitation and hydrophobic interaction chromatography and converted to HAMLET by partial unfolding and binding to oleic acid, as previously described [16]. Briefly, native α lactalbumin was dissolved in Tris (10 mM Tris/HCl pH 8.5) and Ca^2+^ was removed by the addition of 3.5 mM EDTA. The partially unfolded protein was applied to a DEAE-Trisacryl M matrix pre-conditioned with oleic acid at pH 8.5 (Sigma Aldrich, St. Louis, MO). The HAMLET complex was eluted with a NaCl gradient. Dialysis was used to remove excess salt and HAMLET was lyophilized and stored at −20°C. The purity of each HAMLET batch was confirmed by SDS PAGE (Nupage, Invitrogen) and activity by quantifying cell death, as described below.

Human milk was obtained from individual donors, after signed informed consent. Each donor was aware that the samples may be used in scientific research. The samples were de-identified and steps were taken to protect the participants' identities. The procedure was approved by the human ethics committee of the Medical Faculty, Lund University, Lund, Sweden.

### Cells

To identify conserved response pathways explaining the broad tumoricidal effects of HAMLET [25] tumor cells differing in tissue origin, oncogene repertoire, cell membrane composition, ion channel expression and growth capacity were used. T-cell lymphoma cells (Jurkat), lung carcinoma (A549), ovarian carcinoma (HeLa) and kidney (A498) carcinoma cells (ATCC, Manassas, VA) were cultured in RPMI-1640 with non-essential amino acids (1∶100), 1 mM sodium pyruvate, gentamicin (50 µg/ml, Gibco, Paisley, UK), and 5% (A549 and Jurkat) or 10% (HeLa and A498) fetal calf serum (FCS, Gibco), respectively. Healthy, differentiated human renal epithelial cells (HRTEC) in primary culture were kindly provided by Prof. D. Karpman (Lund University, Lund, Sweden) after ethical approval from the Medical Ethics Committee of the Lund University Medical Faculty (decision number LU 456–96). These cells have previously been shown to be resistant to the lethal effects of HAMLET [16]. HRTECs were cultured in DMEM/F12 with 15% FCS (as in [26]) Primary RPTEC cells (human renal proximal tubule epithelial cells) were purchased from Lonza (Basel, Switzerland) and cultured in DMEM-F12 supplemented with NEAA, sodium pyruvate, gentamicin, glutamax and 15% FBS (Gibco).

### Cell Death Assays

Carcinoma cells were detached from cell culture flasks with Versen (0.2 g EDTA in 200 ml H_2_O and 800 ml PBS) washed with PBS and resuspended in serum-free RPMI-1640. Cells were seeded at a density of 50.000/well in a 24-well plate and allowed to adhere overnight in growth medium. HAMLET dissolved in PBS was incubated with cells in serum-free medium and FCS was added after 1 hour. Jurkat cells in suspension (>80% viable) were mixed with HAMLET at 37°C at a density of 10^6^/ml in serum-free media. All cell death measurements were carried out three hours after HAMLET addition, unless otherwise stated. Cell death was quantified by trypan blue exclusion (Chroma Gesellschaft Schmid & Co) by counting of at least 300 cells/sample or by measuring ATP levels (ATPlite Kit, PerkinElmer, Infinite F200, Tecan). Light images were captured using the HoloMonitor™ M2 digital holographic microscope (Phase Holographic Imaging AB, Lund, Sweden). Trypan blue is a classical vital dye, reflecting the loss of membrane integrity in dying cells. ATP is widely used as a surrogate biochemical marker for viability, based on the assumptions that living cells produce ATP and it is indispensable for cellular life [27]. HAMLET treated cells have previously been examined for evidence of programmed cell death, including apoptosis and autophagy [28], [29]. While caspases and autophagy are activated in HAMLET treated cells, inhibition of these pathways does not rescue the cells from HAMLET-induced death. Therefore apoptotic and autophagic parameters are unlikely to explain the HAMLET-induced effects and were not examined in this study.

### Intracellular Ion Concentrations and Ion Fluxes

The relative, free intracellular concentrations of Ca^2+^ ([Ca^2+^]_i_) and Na^+^ ([Na^+^]_i_) were estimated using the calcium indicator Fluo-4 NW and the sodium fluorophore CoroNa Green, respectively (Invitrogen). For Ca^2+^ measurements, A549 cells were given fresh medium with Fluo-4 NW (5 µM) and incubated at 37°C for 30 min, and subsequently treated as indicated. Fluo-4 fluorescence was measured at 535 nm after excitation at 485 nm using a fluorescence plate reader (TECAN infinite F200, Tecan Group, Switzerland). The Ca^2+^ ionophore A23187 (1 µM, Invitrogen) was used as a positive control. Relative [Na^+^]_i_ was measured in Jurkat cells by loading the cells with the sodium indicator CoroNa Green (Invitrogen) at 10 µM for 30 min. For estimation of K^+^ fluxes, the FluxOR™ potassium ion channel assay (Invitrogen) was used according to the manufacturer’s instructions. Briefly, cells were incubated with FluxOR™, which is a Tl^+^ indicator. An increase in fluorescence signal corresponds to an influx of Tl^+^, indicating opening of potassium channels, which was measured at 535 nm after excitation at 485 nm in the TECAN infinite plate reader. K^+^ fluxes and [Ca^2+^]_i_ were additionally analyzed by confocal imaging. Images were captured every 10 s for 5 min on a LSM510 META confocal microscope (520 nm emission after excitation at 488 nm).

### Patch Clamp Measurements

#### Solutions

The standard extracellular solution contained (in mM): 150 NaCl, 6 CsCl, 2 MgCl_2_, 1 CaCl_2_, 10 HEPES, 10 glucose and pH was adjusted to 7.4 using NaOH. The pipette solution for standard measurement contained (in mM): 20 CsCl, 100 Cs-aspartate, 1 MgCl_2_, 0.08 CaCl_2_, 10 HEPES, 10 1,2-bis(2-aminophenoxy)ethane-N,N,N’,N’-tetraacetate (BAPTA), 4 Na_2_-ATP and pH was adjusted to 7.2 using CsOH. The free calcium concentration of this solution is ∼200 nM. For estimation of the relative permeability of monovalent cations the extracellular solution contained: (in mM) 20 CsCl, 130 XCl, 10 HEPES and 10 glucose where X represents the respective monovalent cation (Na^+^, K^+^, or Cs^+^). All solutions were adjusted to pH 7.4 using Tris. The pipette solution for selectivity measurements was: (in mM) 100 Cs-aspartate, 20 XCl, 10 HEPES and 10 glucose, with X = Na^+^, K^+^, or Cs^+^4 Na_2_-ATP and 5 ethylene glycol tetraacetic acid (EGTA) with X = Na^+^, K^+^, or Cs^+^. pH was adjusted to 7.2 using Tris. To maintain ionic composition, HAMLET, α-lactalbumin and oleate were directly dissolved into recording solutions.

#### Electrophysiological recording

For patch clamp measurements, A549 cells where seeded on round coverslips 24–48 h before measurement. All measurements were conducted at room temperature with constant perfusion. Whole cell currents were measured using an EPC10-USB amplifier (HEKA Elektronik, Lambrect, Germany), employing ruptured patches. Pipette resistance was 6.5±0.4 MΩ in asymmetrical solutions. Capacitance and series resistance were recorded continuously and 65% of the series resistance was electronically compensated to reduce voltage errors. As reference electrode, Ag-AgCl electrode was used under standard conditions while a 3 M KCl agar bridge was used for selectivity measurements. All measurements where performed using a 400 ms linear voltage ramp from −100 mV to +100 mV. The protocol was initiated by a 50 ms prepulse at −100 mV and followed by 1550 ms at –30 mV. All data were filtered 2.9 kHz and sampled 1 kHz.

#### Calculation of the relative permeability

Due to the relatively narrow window of time from HAMLET stimulation to seal rupture (presumably reflecting membrane perturbation by HAMLET, see Results), relative permeability ratios were calculated from the absolute reversal potentials (V_rev_) using the equation [30]:
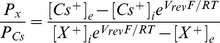
where P_X_ represents gives the permeability of the given ion [X^+^]_i_, [X^+^]_e_ represents the intracellular and extracellular concentrations, and F, T, R has their normal values. The relative Ca^2+^ permeability was calculated as [30]:



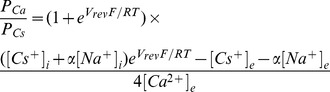
in which α = P_Na_/P_Cs_.

Before calculations, V_rev_ was corrected for liquid junction potentials [31] (V_LJ_) as:
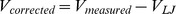



V_LJ_ was calculated using the built-in JPCalcW software in Clampex7 (Axon Instruments, USA).

### Confocal Microscopy

For confocal microscopy, cells were cultured overnight on 8-well chamber slides (Nalge Nunc A/S, Roskilde, Denmark). After the respective experimental procedures, cells were fixed in 3.7% paraformaldehyde, nuclei and plasma membrane were stained with Draq5 (eBiosciences) and Alexa-Fluor 488-labeled wheat germ agglutinin (Invitrogen) and examined in a LSM510 DUO confocal microscope using a 63× oil immersion objective (Carl Zeiss, Jena, Germany). The 488 nm Argon laser line was used to excite Alexa-Fluor 488 fluorescence, which was detected using a band pass filter from 505 to 530 nm. Alexa-Fluor 568 was excited using the HeNe543 nm laser, and fluorescence was detected using a band-pass filter from 560 to 615 nm. The 633 nm HeNe laser was used to excite Draq5, and fluorescence was measured using a 650 long-pass filter. For ion channel inhibition, cells were pre-incubated with medium containing inhibitors for 30 minutes and treated with 35 µM HAMLET (3.5 µM Alexa-Fluor 568-labeled HAMLET (Invitrogen, Carlsbad, CA) and 31.5 µM unlabeled HAMLET) in serum-free medium. For live cell imaging, cells were maintained at 37°C, nuclei were stained with Hoechst 33342 (Invitrogen) and Alexa-Fluor 568-labeled HAMLET was added in serum-free medium. The cells were kept at 37°C, 5% CO_2_ and images were collected regularly by confocal microscopy, using a LSM510 META (Carl Zeiss, Jena, Germany).

### Transcriptomic Analysis

For the microarray analysis, 200.000 A549 cells/well were allowed to adhere overnight on a 6-well plate. After 1 h of HAMLET treatment (21 µM), attached as well as floating cells were lysed and RNA was extracted using the RNeasy Mini Kit (QIAGEN). The samples were sent to AROS Applied Biotechnology (Århus, Denmark) for analysis and run on Human Genome U219 Array Plate according to standard Affymetrix protocols. Data analysis was carried out using R and Bioconductor (http://www.r-project.org). The raw data was normalized using RMA (Irizarry et al., 2003) in which raw intensities are background-corrected, log2 transformed and then normalized using quantiles. A linear model is fitted to the data to obtain expression value for each probe set. The normalized data was found to be of excellent quality with high replicate correlation (>0.99) and NUSE (normalized unscaled errors) values close to 1 (range 0.994–1.008). To derive differentially expressed genes a linear model was fitted using the Bioconductor limma package and genes with empirical Bayes adjusted p-values <0.05 and log2 fold changes >1 were considered differentially expressed and were functionally characterized using the Database for Annotation, Visualization and Integrated Discovery (DAVID; (Dennis et al., 2003) and Ingenuity Pathway Analysis. For the extended microarray analysis, gene expression was assessed by whole genome Illumina microarrays (HumanHT-12 Expression BeadChip). Data was normalized using cross-correlation [32]. Genes with a Benjamini-Hochberg adjusted p-value <0.05 and log2 fold change ≥1.2 in tumor cells and ≥2.0 in normal, differentiated cells at any time point were regarded as differentially expressed. All microarray data were registered into NCBI's Gene Expression Omnibus (GEO) database (http://www.ncbi.nlm.nih.gov/projects/geo) with accession number GSE23772.

### Western Blots and Cytokine Quantification

For Western blots, 200.000 cells were allowed to adhere overnight in a 6-well plate, exposed to different experimental conditions and lysed in M-PER lysis buffer (Pierce, Rockford, IL) containing Complete Protease Inhibitor Cocktail and PhosSTOP phosphatase inhibitor cocktail (both from Roche, Mannheim, Germany). The lysates were cleared by centrifugation and protein concentrations were measured using the DC Protein Assay (Bio-Rad Laboratories, Hercules, CA) on a Tecan Infinite plate reader. Equal amounts of protein were separated by SDS-PAGE on 4–12% Bis-Tris gels (Invitrogen) and blotted onto PVDF membranes (GE Healthcare). Membranes were saturated with BSA (GAPDH), nonfat dry milk (phospho-p38 MAPK, p38 MAPK, phospho-ERK1/2, ERK1/2, ATF6, phospho-eIF2α) or Sat-1 and Sat-2 (α-lactalbumin) and incubated with anti-bovine α-lactalbumin (1∶500, Bethyl Laboratories, Montgomery, Texas), anti-p38 MAPK, anti-phospho-(Thr180/Tyr182)p38 MAPK, anti-ERK1/2, anti-phospho-(Thr202/Tyr204)-ERK, anti-phospho-eIF2α (Ser51) (all 1∶500–1000, Cell Signaling Technology, Danvers, MA), anti-ATF6 (1∶1000, IMG-273, Imgenex) or anti-GAPDH (1∶3000–5000, Novus Biologicals) antibodies followed by horseradish peroxidase-conjugated anti-rabbit (1∶1000, DakoCytomation, Glostrup, Denmark), anti-goat (1∶1000, Sigma Aldrich) or anti-mouse (1∶40.000–50.000, Novus Biologicals) secondary antibodies for staining. Bound antibodies were detected with ECL Plus Western Blotting Reagent (GE Healthcare, Little Chalfont, UK) and GelDoc equipment (Bio-Rad Laboratories, Hercules, CA). To control for equal loading, membranes were stripped with Restore Western Blot Stripping Buffer (Pierce), blocked and reprobed with new antibodies. For siRNA experiments, equal volumes of lysates were separated by SDS-PAGE on 4–12% Bis-Tris gels (Invitrogen) and blotted onto PVDF membranes.

MAPK phosphorylation was analyzed on a Human Phospho-MAPK array (Proteome Profiler Array, R&D Systems, Minneapolis, MN) as per the manufacturer’s instructions. Band and spot intensities were quantified using ImageJ [33]. Cytokine quantification (IL-6, IL-8, TNF-α) was performed on an IMMULITE 1000 immunoassay system (Siemens Diagnostics, Deerfield, IL).

### Inhibitors and RNAi

Pharmacologic ion channel inhibitors are widely used as tools to define the function of specific ion channel classes. Amiloride inhibits several Na^+^-carrying channels and transporters, incl. ENaC type Na^+^ channels, Na^+^/H^+^ exchangers, and Na^+^/Ca^2+^ exchangers. Barium chloride (BaCl_2_) blocks many types of potassium channels. Gadolinium chloride (GdCl_3_) is a general inhibitor of mechanosensitive channels and tetrandrine inhibits large conductance Ca^2+^ activated potassium channels. Ruthenium Red is a broad inhibitor of many cation channels including intracellular Ca^2+^ release channels. Amiloride (1 mM), BaCl_2_, (1 mM), Ruthenium Red (30 µM), tetrandrine (10 µM) and GdCl_3_ were from Sigma Aldrich. For p38 MAPK inhibition, SB202190 (20 µM, Sigma Aldrich) or BIRB796 (10 µM, Axon Medchem) were used.

For RNA interference, FlexiTube siRNA Premixes against MAPK11 (SI00606053), MAPK14 (SI00300769) and All Star Negative Control siRNA (SI03650318) from QIAGEN (Hilden, Germany) were used. A549 cells were forward transfected using a 25 nM final siRNA concentration in 24-well plates. For p38 MAPK, knockdown was examined by Western blot and RT-PCR 48 h after transfection.

### Statistical Analysis

Repeated measures ANOVA or two-sided Students *t*-test were applied as relevant and were performed with InStat software (version 3.06, GraphPad, San Diego, CA). Test for normality was done using InStat, using the Kolmogorov-Smirnov test. For all experiments, a p-value <0.05 was considered significant. The error bars in all graphs represent SEMs from at least three independent biological replicates.

## Results

### Na^+^, K^+^and Ca^2+^ Fluxes Induced by HAMLET in Tumor Cells

**Figure 1:**
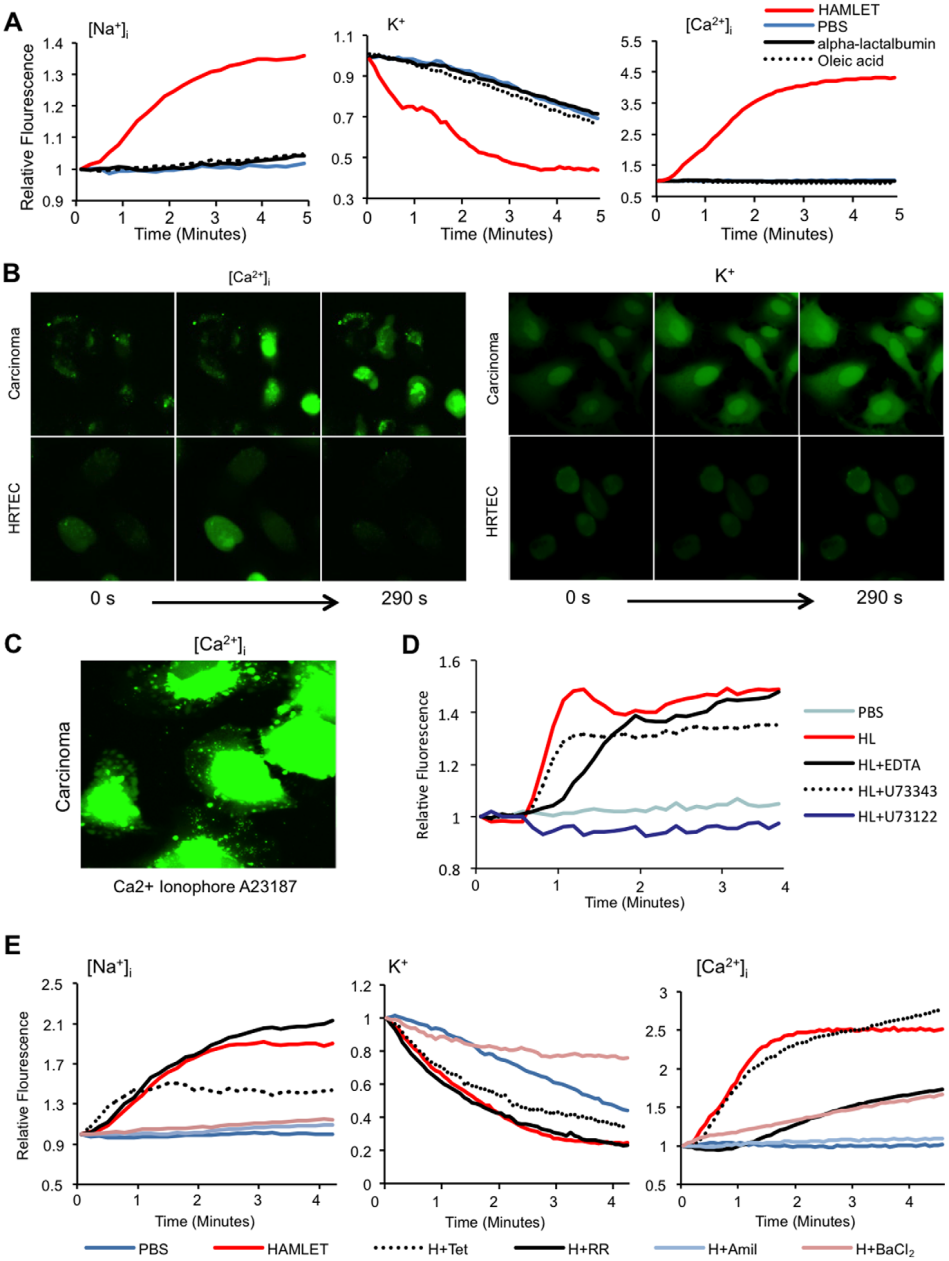
HAMLET alters intracellular ion concentrations in tumor cells. (A) Estimates of the relative free, intracellular concentrations of Na^+^, K^+^, and Ca^2+^ ([Na^+^]_i_, [K^+^]_i_, and [Ca^2+^]_I_, respectively) were obtained in A549 cells (K^+^ and Ca^2+^) and Jurkat cells (Na^+^) by fluorescence spectrometry using CoroNa Green, FluxOR and Fluo-4. Rapid ion fluxes were detected and these effects were HAMLET specific, as α-lactalbumin, oleic acid or PBS had no effect. (Means of four experiments, p<0.05 (Students t-test at t = 2 minutes) (B) Difference in HAMLET-induced ion fluxes between tumor cells and healthy cells. Ca^2+^ and K^+^ fluxes are visualized by real time confocal imaging of A549 lung carcinoma cells and HRTEC healthy, differentiated kidney cells, loaded with the Ca^2+^ and K^+^ fluorophores and treated with HAMLET (35 µM) for up to 290 seconds. Representative figures from three experiments are shown. (C) Calcium ionophore (A23187, 1 µM) response in A549 lung carcinoma cells loaded with Fluo-4. Representative figure from three experiments. (D) Inhibition of intracellular calcium release from ER reduces Ca^2+^ fluxes. A549 cells were pretreated with a PLC-inhibitor (10 µM, U73122), its inactive analogue (10 µM, U73343) or EDTA (1 mM) for 30 minutes and treated with HAMLET as indicated. Means of three experiments. (E) Amiloride (Amil, 1 mM) inhibited Na^+^ and Ca^2+^ fluxes in A549 cells (Ca^2+^) and Jurkat cells (Na^+^) induced by HAMLET (marked as H in figure). BaCl_2_ (1 mM) inhibited the Na^+^, K^+^ and Ca^2+^ fluxes. Cells were pre-loaded with the respective fluorophores, pretreated with inhibitors (30 minutes) and challenged with HAMLET (35 µM) for up to 5 minutes. Effects of amiloride on K^+^ fluxes could not be determined due to auto fluorescence. Ruthenium red (RR, 30 µM) reduced Ca^2+^ fluxes while tetranidrine (Tet, 10 µM) had no significant effect.

Changes in intracellular ion concentrations induced by HAMLET (35 µM) in tumor cells were characterized by fluorometry (Figure 1A) and real time confocal imaging (Figure 1B). HAMLET triggered a rapid increase in [Na^+^]_i_ in Jurkat cells preloaded with the Na^+^ fluorophore CoroNa Green. Opening of a plasma membrane K^+^ transport pathway in response to HAMLET was recorded in A549 lung carcinoma cells using the FluxOR™ assay, which monitors the influx of thallium as a surrogate marker for K^+^ [34]. Given the driving force for K^+^ flux across the plasma membrane this corresponds to K^+^ efflux, assuming that the transport pathway is a channel (see Discussion). HAMLET also triggered a rapid and sustained increase in [Ca^2+^]_i_ in Fluo-4AM preloaded lung carcinoma cells (Figure 1A). In contrast, native α-lactalbumin or oleic acid did not induce Na^+^, K^+^or Ca^2+^ fluxes (Figure 1A).

The [Ca^2+^]_i_ and K^+^ ion fluxes were also visualized using confocal microscopy and shown to occur in almost all cells (Figure 1B). These rapid ion fluxes were not observed by confocal microcopy in healthy, differentiated cells in primary culture (HRTEC). Confocal imaging of Fluo-4 loaded cells revealed a difference in kinetics and magnitude between responses to HAMLET or the Ca^2+^ ionophore A23187 (Figure 1C), suggesting that they work by distinct mechanisms. Ca^2+^-chelation with EGTA had little effect on the increase in [Ca^2+^]_i_, suggesting that the involvement of extracellular Ca^2+^ was minimal (Figure 1D). However, inhibition of InsP3-gated ER Ca^2+^ channels with U73122 essentially abolished the increase in [Ca^2+^]_i_, implying that it originated mainly from intracellular stores (Figure 1D, [35]).

### HAMLET-induced Ion Fluxes are Amendable to Ion Channel Inhibitors

A panel of well-characterized ion channel inhibitors was used to further investigate the HAMLET induced ion fluxes. The change in [Na^+^]_i_ in Jurkat cells was inhibited by amiloride and BaCl_2_, the K^+^ flux in lung carcinoma cells by BaCl_2_ and the Ca^2+^ flux by amiloride and BaCl_2_ (Figure 1E). The effect of amiloride on the K^+^ flux could not be tested, due to autofluorescence. The molecular nature of the HAMLET-activated current was further investigated using the broad-spectrum Ca^2+^ channel inhibitors, Ruthenium red and tetrandrine, none of which significantly affected the current (Figure 1E).

### HAMLET Activates a Nonselective Whole Cell Cation Current in Carcinoma Cells

**Figure 2:**
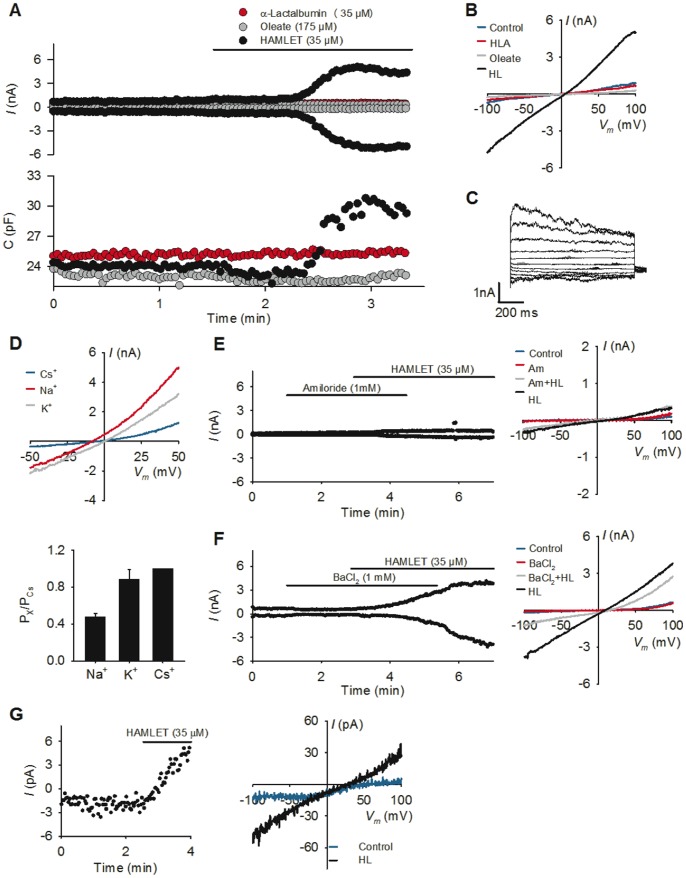
Whole cell currents activated by HAMLET in lung carcinoma cells. HAMLET-induced currents were recorded in A549 cells, by whole cell patch clamp analysis, with the exception of data in G, which were obtained in inside-out patches. (**A**) *Top panel:* Currents were measured using the described ramp protocol and currents measured at +100 mV and −100 mV are depicted as a function of time for three representative and independent experiments stimulated with α-lactalbumin, oleate or HAMLET, the presence of which in the superfusate is indicated by the top line. *Lower panel:* Cell capacitance as a function of time. Same experiments as in top panel. (**B**) Same experiments as in A, showing the current- voltage relationship of cells following the indicated stimulation. The control trace represents the current-voltage relationship before exposure to HAMLET (n = 7). (**C**) Using a 1 s protocol stepping from −100 mV to +100 mV in 20 mV increments, time dependent characteristics of the HAMLET activated current were investigated. Note the clear time dependent inactivation at strongly depolarized potentials. n = 3. (**D**) Selectivity profile of HAMLET activated currents. *Top panel*: Reversal potentials of HAMLET activated currents where estimated from individual experiments as described in Materials and Methods. *Lower panel:* Permeability ratios for Na^+^ and K^+^ relative to Cs^+^, calculated as described. n = 3–5 (**E**) Amiloride inhibits HAMLET activated currents. *Left panel:* Current magnitude measured at −100 mV and +100 mV. The presence of amiloride (1 mM) and HAMLET in the superfusate is indicated by the top lines. Compare the current with that in the absence of amiloride in (A). *Right panel:* Current-Voltage relationship from same experiment as in the left panel. n = 4. (**F**) BaCl_2_ partially blocks the inward current. *Left panel:* Current magnitude measured at −100 mV and +100 mV. The presence of BaCl_2_ (1 mM) and HAMLET in the superfusate is indicated by the top lines. *Right panel:* Current-Voltage relationship from the same experiment as in left panel. Note the strong inhibition of the inward current compared to the outward current. n = 3. (**G**) HAMLET stimulates current activation in inside-out patches. *Left Panel:* Currents in inside-out patches were followed at the holding potential of the described ramp protocol and plotted as a function of time. The presence of HAMLET in the perfusion solution is indicated above. *Right panel:* Current voltage relationship measured in inside-out patches in the presence and absence of HAMLET. Same experiments as in the left panel. n = 3.

The observed changes in intracellular ion concentrations were indicative that HAMLET activated a non-selective ion channel. This hypothesis was confirmed by whole cell patch clamp experiments (Figure 2). HAMLET (35 µM) activated a whole cell current, which reached a magnitude of 2.74±0.88 nA within 1.43±0.13 min. The current exhibited a rather linear current-voltage-relationship (Figure 2A,B) and a clear time-dependent inactivation at depolarized potentials (Figure 2C). Initial current activation exhibited a delay of 0.88±0.13 min from HAMLET application. In contrast, α-lactalbumin or oleate, were inactive, emphasizing the need for the HAMLET complex to activate the cell current (Figure 2A–B). The reversal potential (V_rev_) in Cs-based solutions was −5.1±0.99 mV, corresponding to the calculated Nernst potential 4.85 mV while V_rev_ was −8.3±1.3 mV and 2.4±2 mV in Na^+^ and K^+^ based solutions, respectively, giving a permeability ratio of P_Cs_ (1)>P_K_ (0.88±0.1)>P_Na_ (0.48±0.03) (Figure 2D). As HAMLET was insoluble in high Ca^2+^ solutions and the time between application and cell rupture due to membrane incorporation (see later) did not allow for solution exchange, P_ca_ was not measurable. However, in Ca^2+^ free medium, the rate of the HAMLET induced increase in [Ca^2+^]_i_ was reduced, suggesting at least a minor Ca^2+^ permeability of the channel. The current was inhibited by the non-specific cation channel inhibitor amiloride (Figure 2E), and was blocked in a voltage-dependent manner by BaCl_2_ (Figure 2F).

The characteristics of the HAMLET-induced current are at variance with the biophysical and pharmacological characteristics of most widely expressed cation channels such as the transient receptor potential (TRP), epithelial Na^+^ channel (ENaC) and various K^+^ channel families. While the biophysics and pharmacology showed some similarity to the cyclic-nucleotide-gated (CNG) channels [36], which are expressed and functional in A549 cells [37], the inclusion of 8-Br-cGMP (1 mM) in the pipette solution activated a current with different biophysical properties. Furthermore, a CNG-channel inhibitor did not affect the HAMLET-induced current (L-cis-diltiazem, LCD (200 µM, Figure S1) or the death, confirming that CNG channels are not major HAMLET targets (Figure S1E).

In a further attempt to identify the channel, a broad range of methods was applied. By gene expression analysis (see below), the ion channel repertoire of tumor cells and healthy cells was compared. The expression of around 400 ion channel genes was surveyed in exponentially growing lunch carcinoma (A549) and kidney carcinoma (A498) cells using Illumina whole genome arrays. The ion channel mRNA repertoire was also compared to that of Human Renal Proximal Tubular Epithelial Cells (HRPTEC) in primary culture. As expected, only a fraction of the ion channels in the human genome was expressed in each tumor cell line (around 5%, defined as a 3-fold change above the background). The cell lines expressed one constitutively active Na^+^ channel (SCNN1A) and one voltage-gated Na^+^ channel (SCN3A) as well as about 200 voltage-gated K and Ca^2+^ channels with no major differences in the number or level of expressed ion channel genes between carcinoma cells and normal cells**.** Furthermore, an ion channel ligand library containing 69 ion channel blockers and openers was used for further characterizing and identifying ion channels in individual cells affecting the response to HAMLET. However, no single inhibitor, other than those previously identified (Amiloride and BaCl_2_) was able to reproducibly alter HAMLET’s effect. Thus, using these technologies, no conclusive target was identified, further indicating that the channel activated by HAMLET exhibits some unusual properties, perhaps reflecting a novel subunit composition, cancer-cell specific alternative splicing or a HAMLET induced channel (see Discussion).

Interestingly, application of HAMLET increased cell capacitance from 24.5±1.1 pF to 30.4±1.5 pF (n = 7, p<0.001) (Figure 2A, lower panel). The temporal pattern correlated closely with that of the induced current (Figure 2A, upper panel). The current was also activated when HAMLET was applied directly to inside-out membrane patches, further supporting a direct effect at the level of HAMLET at the plasma membrane (Figure 2G). Collectively, the results strongly suggest that HAMLET’s membrane-perturbing effects may be the trigger for channel activation.

### Ion Channel Inhibition Prevents HAMLET-induced Cell Death

**Figure 3:**
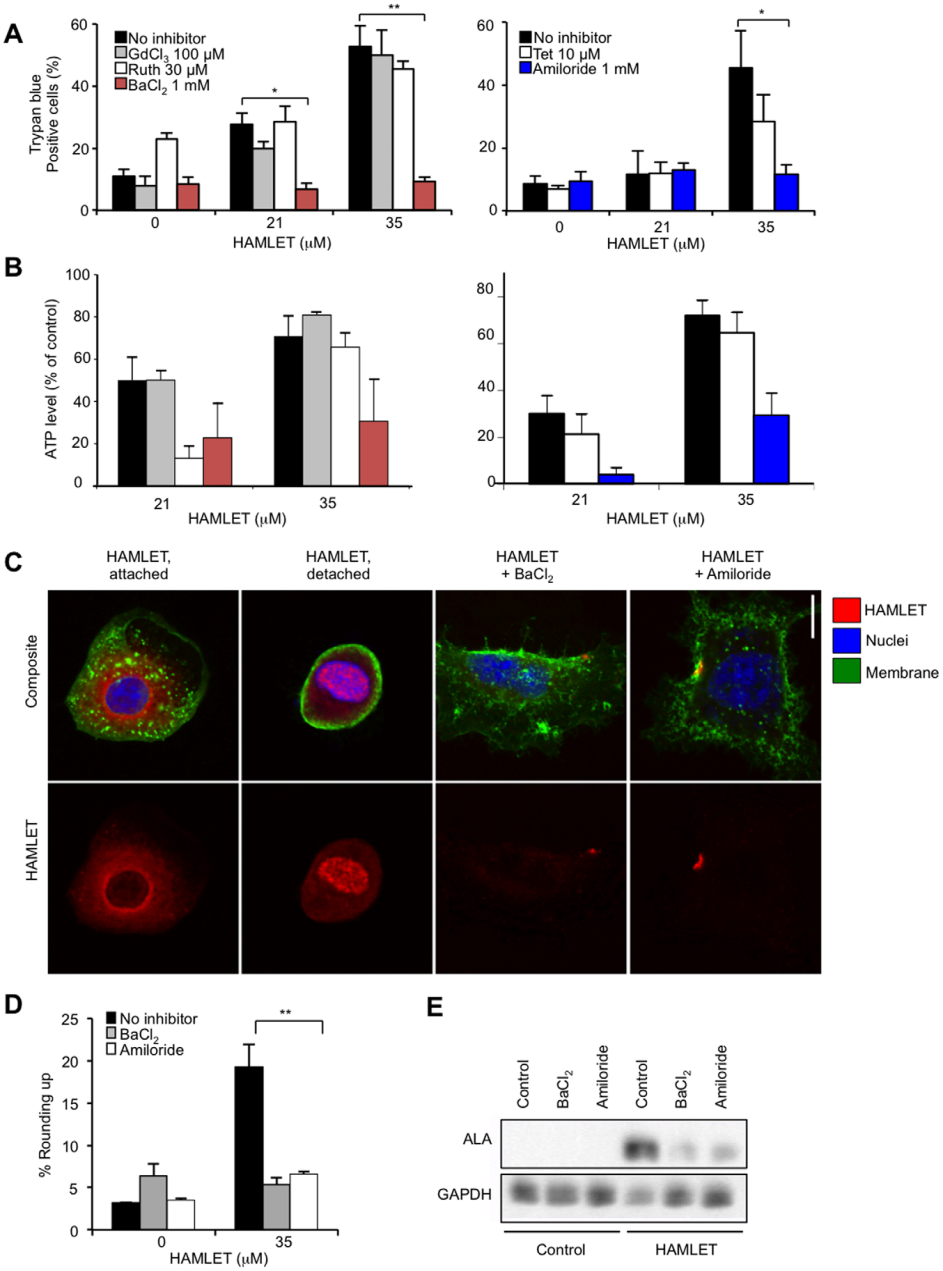
Ion channel inhibitors rescue carcinoma cells from death and block HAMLET uptake and morphologic change. (A, B) Viability of lung carcinoma cells after exposure to HAMLET (21 or 35 µM, 3 h), quantified by trypan blue exclusion and ATP levels. Amiloride or BaCl2 inhibited the tumoricidal effect of HAMLET but Ruthenium Red and tetrandrine showed no effect (means+SEMs, two to three experiments per condition). (C) Internalization of Alexa-568 fluor labeled HAMLET by tumor cells (35 µM, 1 hour), visualized by high magnification (x63) confocal microscopy. HAMLET was localized to peri-nuclear and nuclear regions in tumor cells. Amiloride or BaCl_2_ inhibited internalization, leaving HAMLET associated with the cell surface. HAMLET was labeled red (Alexa-568), nuclei blue (Draq5) and membranes were labeled green (WGA). Scale bar = 10 µM. (D) BaCl_2_ and amiloride prevented changes in carcinoma cells morphology in response to HAMLET (Mean of two images in one experiment+SEMs). Rounding up signifies the shift from an adherent, extended to a rounded morphology. (E) Western blot of cell lysates confirming the reduction in cell-associated HAMLET by amiloride and BaCl_2_ (35 µM, 1 hour, detected with anti-α-lactalbumin antibodies, *ALA*). GAPDH was used as a loading control.

The ion channel inhibitors were further used to examine whether ion fluxes are important for the tumoricidal activity of HAMLET. Lung carcinoma cells were pretreated for 30 min with amiloride, BaCl_2_, Ruthenium red or tetrandrine, and exposed to HAMLET (21–35 µM, 3 h). Changes in cell viability were quantified by two techniques. The trypan blue exclusion assay detects the accumulation of vital dye secondary to changes in membrane integrity in dying cells [38]. Cellular ATP levels are widely used as a surrogate biochemical marker for viability [27]. The rapid, dose-dependent loss of viability in response to HAMLET was detected by both techniques with similar kinetics (Figure 3A–B). Inhibition of ion fluxes by amiloride or BaCl_2_, reduced cell death from about 50% to <20% (p<0.05) (Figure 3A–B).

The reversal of cell death by amiloride and BaCl_2_ was confirmed in ovarian carcinoma cells (HeLa cells) and lymphoma cells (Jurkat cells), under the same experimental conditions (Figures S2A, B and S3A, B). Furthermore, the rescue effect of amiloride or BaCl_2_ on carcinoma cells was sustained. About 80% of the tumor cells had died after 24 h of HAMLET treatment (35 µM) but cells pretreated with amiloride or BaCl_2_ remained viable (9% and 34% cell death, respectively, Figure S3C) and combining both ion channel inhibitors completely rescued the cells from HAMLET-induced death (Figure S3D).

To further address if extracellular Ca^2+^ contributes to the cell death response, the cytotoxic effect of HAMLET was tested in Ca^2+^-free medium. While the tumoricidal effect of HAMLET was unchanged in the absence of Ca^2+^, combining amiloride and BaCl_2_ still prevented HAMLET-induced death (Figure S3D). The tumoricidal effect of HAMLET was also unchanged by U73122, a phospholipase C inhibitor, which consequently blocks Ca^2+^ release from the ER via the inositol trisphosphate (InsP3) receptor channel (Figure S3E). In congruence with their lack of effect on [Na^+^]_i_ and [K^+^]_i_, neither Ruthenium red nor tetranidrine rescued lung carcinoma, ovarian carcinoma or lymphoma cells from HAMLET-induced cell death (Figure 3A, S2 and S3).

Collectively, these results suggest that HAMLET triggers rapid [Na^+^]_i_ and [K^+^]_i_ fluxes by activation of an amiloride- and Ba^2+^-sensitive, nonselective cation channel and that these effects are essential for HAMLET-induced cell death.

### Ion Channel Inhibition Prevents HAMLET-induced Morphological Change and HAMLET Internalization

HAMLET treated carcinoma cells internalize large amounts of HAMLET [39]. To address if ion fluxes are involved in the internalization process, the internalization of Alexa 568-labeled HAMLET by lung carcinoma cells was quantified in the presence or absence of Amiloride or BaCl_2,_ (Figure 3C, S4). Internalization was markedly reduced by amiloride or BaCl_2,_ and the inhibitory effect was confirmed by western blots of whole cell extracts (35 µM HAMLET, 1 h, Figure 3E). In parallel, the change in morphology, which characterizes the response to HAMLET, was also reduced (Figure 3C–D). Neither of the ion channel inhibitors had any detectable effect on the morphology of the cells in the absence of HAMLET (not shown).

These results suggest that ion fluxes activated by HAMLET control both HAMLET internalization and the HAMLET-induced changes in tumor cell morphology.

### Ion Fluxes Influence the Transcriptional Response to HAMLET

**Figure 4:**
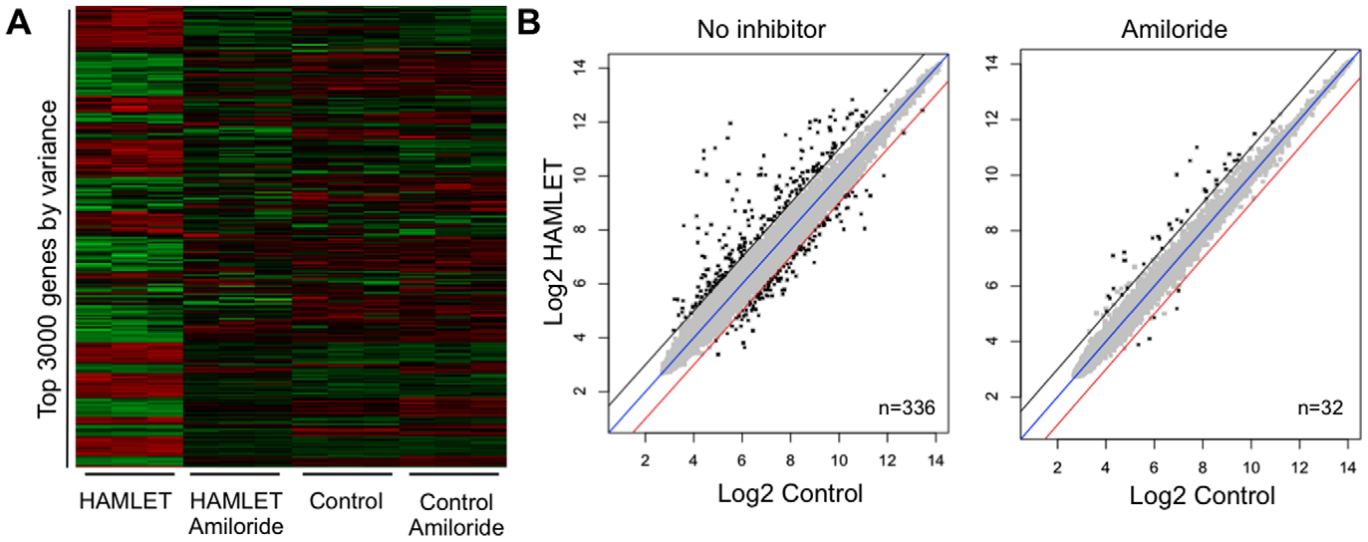
The transcriptomic response to HAMLET requires functional ion channels. Genome wide expression analysis in lung carcinoma cells stimulated with HAMLET in the presence or absence of Amiloride. (A) Heatmap of the normalized expression of top 3000 genes sorted according to variance across all conditions. Amiloride markedly reduced the global transcriptional response to HAMLET but had little effect on control cells. (B) Scatter plots of normalized expression values for all genes present on the array. The number of differentially expressed genes (log2 fold change >1 and FDR-adjusted p-value <0.05, marked in black) was greatly reduced by amiloride.

Gene expression profiling technology is used to define how cellular responses affect transcription and to predict resulting phenotypes, including those involved in cell death [40]. The global transcriptional response to HAMLET was therefore examined, using whole genome arrays, before and after exposure of carcinoma cells to HAMLET (35 µM, 1 h). Hierarchical clustering of the top 3000 genes by variance (Figure 4A) and differential expression analysis detected 336 differentially expressed genes (Adjusted p-value <0.05 and Fold change >1, Figure 4A–B) in response to HAMLET. Main regulated gene categories were involved in cell death, chromatin regulation or ER stress. Strongly regulated pathways were identified by Ingenuity pathway analysis and DAVID. Top scoring pathways were p38 MAPK-signaling (Enrichment Score (ES) = 6.7, n = 15), cell death (ES = 4.7, n = 22) and ER stress (ES = 2.2, n = 8).

To address if ion fluxes control the global transcriptional response to HAMLET (35 µM, 1 h), lung carcinoma and kidney carcinoma cells were pretreated with amiloride and exposed to HAMLET, as described above. Amiloride dramatically reduced the transcriptional response to HAMLET (32 differentially genes remained differentially expressed, p<0.001, Figure 4B) and the p38 MAPK-signaling-, cell death- or ER stress pathways were no longer significantly regulated, suggesting that ion fluxes orchestrate most of the early transcriptional response to HAMLET.

### The p38 MAPK Dependent Death Response to HAMLET

**Figure 5:**
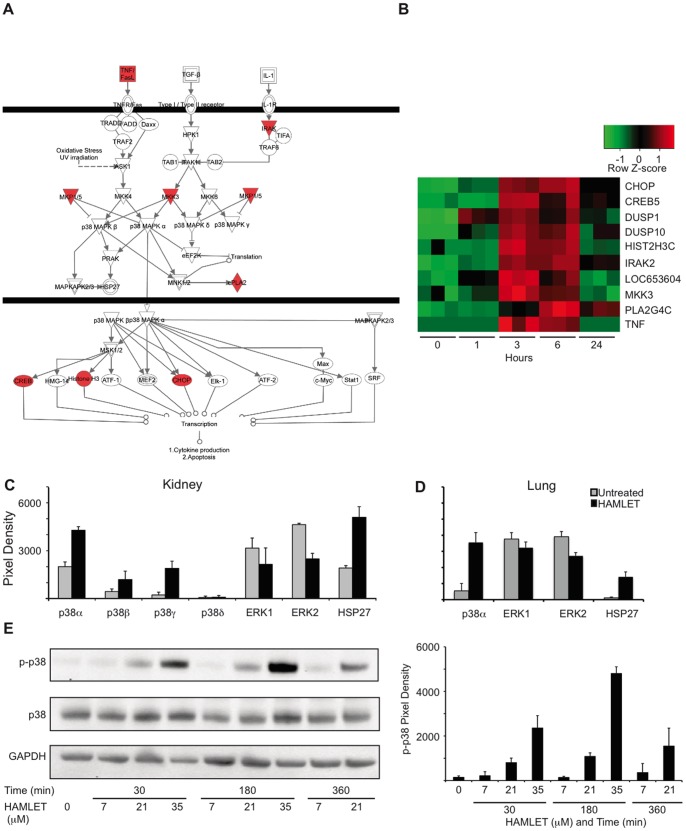
HAMLET activates the p38 MAPK signaling pathway. (A) Transcriptional changes were identified in HAMLET-treated A549 lung carcinoma cells. Three hundred sixty-seven genes showed a minimum log 2-fold change of 1.2 compared to PBS-treated control cells, with a Benjamini-Hochberg adjusted p-value <0.05. Ten genes in the p38 pathway were upregulated (red) three hours after HAMLET treatment (21 µM) of lung carcinoma cells (A549), as marked in the canonical pathway. (B) Heat map (triplicate for each time period) and log2 ratios of differentially expressed genes in the p38 MAPK pathway, 1, 3, 6 and 24 hours after HAMLET exposure. (D, E) Increased phosphorylation of p38α, β, γ or HSP27 and reduced ERK1/2 phosphorylation in kidney (C) and lung (D) carcinoma cells exposed to HAMLET (35 µM, 30 minutes). Membranes with phospho-specific antibodies were probed with protein lysates from HAMLET- or PBS-treated (control) carcinoma cells. Protein phosphorylation was quantified using ImageJ. Data are mean ± SEM of 3 experiments. Full array images are given in Figure S6. (E) Dose- and time-dependent p38 MAPK phosphorylation in response to HAMLET. Lung carcinoma cells were treated with HAMLET and compared to PBS-treated negative controls and whole cell lysates were probed with antibodies specific for phosphorylated p38 MAPK (Thr180/Tyr182). Membranes were stripped and reprobed with total p38 and GAPDH antibody as a loading control. For effects on p38 MAPK signaling in kidney carcinoma cells, see Figure S6.

Marked p38 MAPK-signaling was detected 1 and 6 h after exposure to HAMLET (Figure 5A). The activated p38 MAPK components included MKK3, a direct upstream activator of p38 MAPK along with 8 other p38 MAPK pathway genes (Figure 5B) and two dual-specificity phosphatases (DUSPs; DUSP1 and DUSP10) with log2-fold changes of 1.34 and 1.98, respectively. DUSPs are feedback regulators of MAPK signaling, and are up-regulated when the pathway is active [41]. Upregulated genes downstream of p38 MAPK included CREB5, CHOP and HIST2H3C. After 24 hours, transcriptional activity returned to baseline levels except for IRAK2, PLA2G4C, CHOP and CREB5, which sustained elevated expression. Other activated genes were involved in cell death (ATF3, DDX3X, DDIT3, HSPA1) and chromatin structure (DNAJB3, GADD45A/B, HIST1H1C, KLF2/4/6) regulation [39].

The p38 MAPKs are activated by dual phosphorylation on conserved threonine and tyrosine residues by MKK3/6. Downstream effector proteins include MAPKAPK2 kinase, heat shock protein 27 (HSP27), and the transcription factors ATF2 and CHOP [42]. HAMLET triggered rapid (30 minutes) p38α and HSP27 phosphorylation in lung and kidney carcinoma cells (Figure 5C, D; phosphoarray images given in Figure S6A, B). In addition, p38β and p38γ were phosphorylated in kidney carcinoma cells (Figure 5C). In parallel, a loss of ERK1/2 phosphorylation occurred in both cell types (Figure 5C, D), consistent with a shift from cell proliferation to cell death [43], and with the known inhibitory effect of p38 MAPK on ERK activity after stress stimuli [44]. In contrast, HAMLET had no detectable effect on the phosphorylation of JNK kinase (Figure S6A, B). Phosphorylation of p38 MAPK was confirmed by Western blotting and was dose- and time- dependent (Figure 5E).

**Figure 6:**
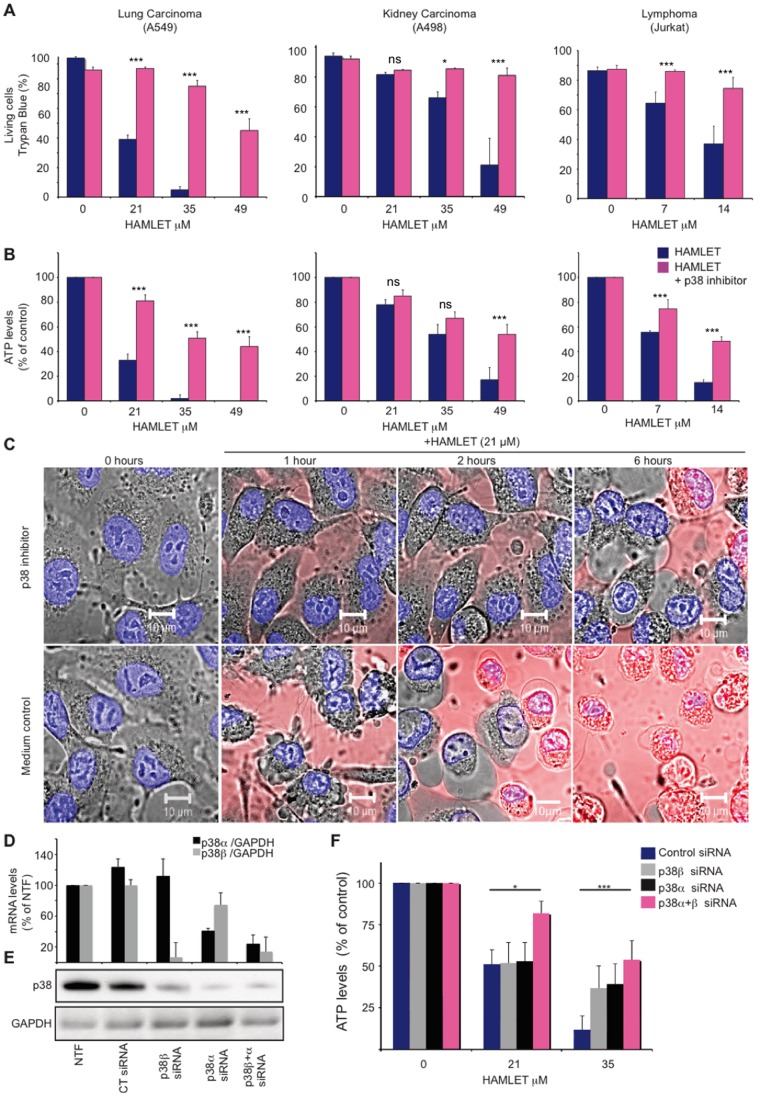
p38 inhibition rescues carcinoma and lymphoma cells from death in response to HAMLET. (A, B) p38 MAPK inhibition (SB202190, 20 µM, 30 minutes pre-incubation) rescued carcinoma (A549 and A498) and T-cell lymphoma (Jurkat) cells from death in response to HAMLET (7–49 µM, 3 h). Viability was quantified by Trypan blue exclusion (A) or as ATP levels (B). Data are means±SEMs for 3 independent experiments. The p38 inhibitor alone had no significant effect on cell death (leftmost bars in each graph). (C) Real-time images of cell morphology after HAMLET exposure, showing that p38 inhibition prevents morphological changes in carcinoma cells (nuclear condensation, rounding up and blebbing). Alexa-Fluor 568-labeled HAMLET is red and the nuclei are blue (Hoechst 33342). (D–E) A549 lung carcinoma cells were transfected using siRNA against p38α and/or p38β or non-targeting siRNA and compared to non-transfected controls (NTF). Relative p38α and p38βmRNA levels are shown (p38/GAPDH, in % of non-transfected cells) as means+SEMs for four independent experiments. Knockdown was also confirmed on the protein level by western blotting against total p38 MAPK (representative blot shown, *p<0.05, ***p<0.001.). GAPDH was used as a loading control. (F) The cytotoxic effect of HAMLET was quantified 48 hours after transfection as a reduction in ATP levels. Data are means+SEM for four independent experiments. *p<0.05, ***p<0.001. For effects of the p38 inhibitor Birb0796, see Figure S6.

To address if p38 MAPK signaling is involved in HAMLET-induced cell death p38α and p38β were inhibited in lung carcinoma, kidney carcinoma and lymphoma cells. SB202190 is a highly specific pyridinyl-imidazole inhibitor and BIRB796 a diaryl urea compound, which bears little structural similarity to SB202190. The tumoricidal response to HAMLET was attenuated by SB202190 (Figure 6A, B) or BIRB796 (Figure S6E, F), accompanied by a marked decrease in p38 MAPK and HSP27 phosphorylation (Figure S6C). By real-time confocal imaging, p38 MAPK inhibition (SB202190) was shown to delay the morphological response to HAMLET for about six hours and internalized Alexa-Fluor labeled HAMLET was delayed (Figure 6C). Furthermore, HAMLET-induced cell death was inhibited by the combined siRNA knockdown of p38α and p38β with knockdown efficacy confirmed by qRT-PCR and western blot (Figure 6D–F). Each siRNA alone was ineffective, however, indicating that each isoform is sufficient to induce cell death.

### Ion Channel Inhibitors Block HAMLET-induced p38 MAPK-phosphorylation in Tumor Cells

**Figure 7:**
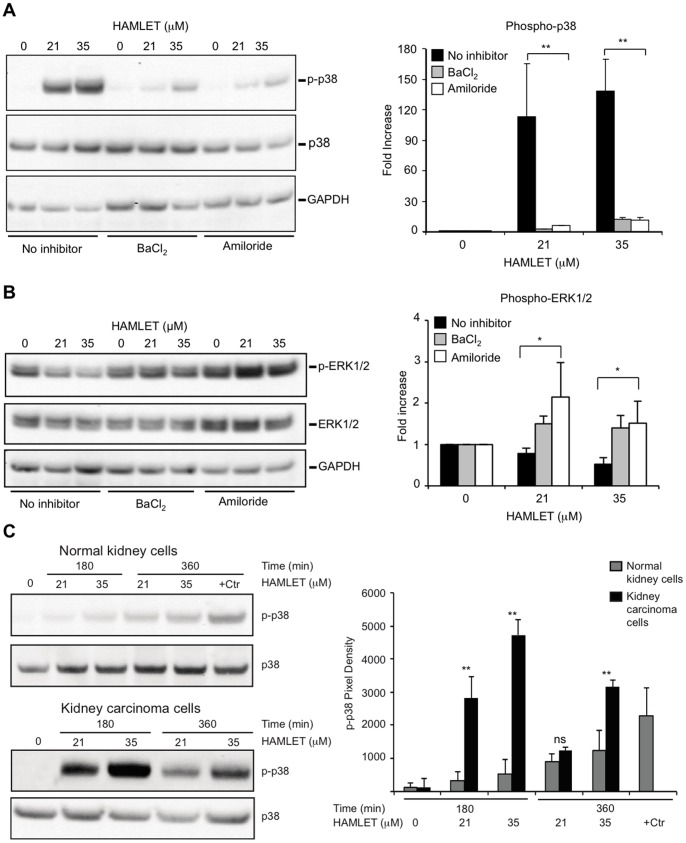
Ion channel inhibitors reduce protein phosphorylation. Amiloride or BaCl_2_ reduced phosphorylation of targets in the p38 signaling Lung carcinoma cells were exposed to HAMLET (21 and 35 µM) for 1 hour, protein lysates were blotted, incubated with antibodies as noted in the figure and quantified using ImageJ (Representative blot,+SEMs of 2–3 independent experiments). (A) Amiloride or BaCl_2_ (1 mM, 30 minutes pretreatment) inhibited HAMLET-induced p38 MAPK phosphorylation (B) Amiloride and BaCl_2_ reverse the suppression of p-ERK1/2 phosphorylation by HAMLET. (C) Difference in phosphorylation of p38 MAPK between normal differentiated cells (HRTEC) and kidney carcinoma cells, visualized by phospho-specific antibodies.

To examine whether ion channels control responses to HAMLET through effects on p38 signaling, tumor cells were pretreated with ion channel inhibitors and p38 MAPK-, and ERK1/2 phosphorylation in response to HAMLET was quantified using phospho-specific antibodies. Amiloride and BaCl_2_ reduced HAMLET-induced p38 MAPK-phosphorylation in lung carcinoma cells (Figure 7A) and in parallel, the reduction in ERK1/2 phosphorylation was reversed (Figure 7B). These results suggest that the p38 MAPK pathway orchestrates the early death response to HAMLET.

#### Normal, differentiated cells survive HAMLET with an innate immune response

Notably, healthy differentiated cells in primary culture (HRTEC) showed minimal increase in dual-phosphorylated (Thr180/Tyr182) p38 MAPK in response to HAMLET treatment, in marked contrast to kidney carcinoma cells (A498), where more than a 40-fold increase in p38 MAPK phosphorylation was detected (Figure 7C). The p38 MAPK pathway was intact in the healthy cells, however, as osmotic shock by 300 mM NaCl (30 min), which was used as a positive control, induced a 19-fold increase in p38 MAPK phosphorylation.

**Figure 8:**
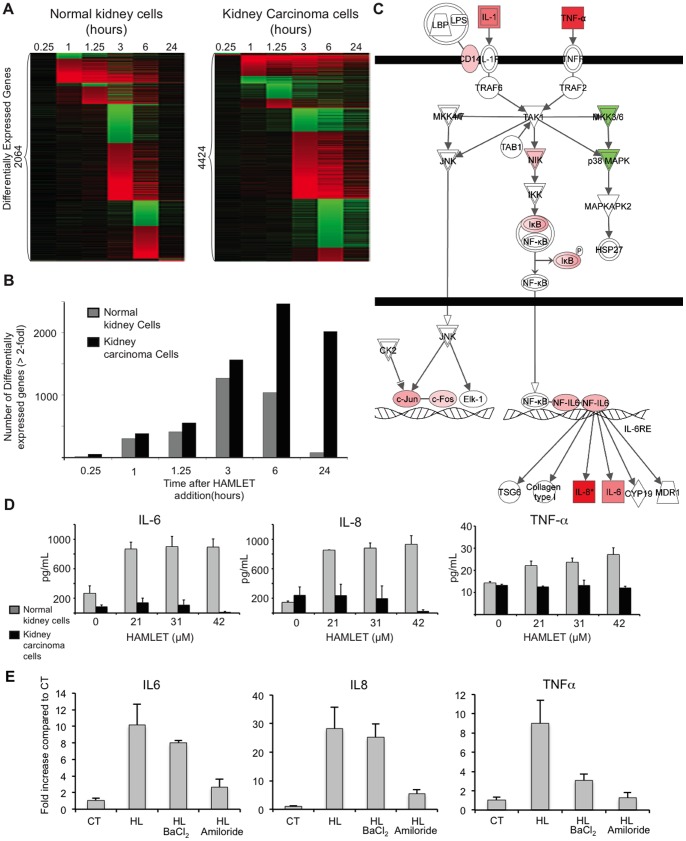
Innate immune response to HAMLET in normal, differentiated cells. (A) The transcriptional response to HAMLET is qualitatively different in normal cells (RPTEC), as shown by the heat map of genes with a log2 fold change >2 at any time point. (B) The number of differentially expressed genes (log2 fold change ≥ 2) was reduced compared to carcinoma cells. (C) Seven innate immunity-related genes are upregulated in normal cells, 75 minutes after HAMLET treatment. Two genes, p38 MAPK and MKK3/6, are downregulated. (D) Confirmation of the innate immune response to HAMLET. Elevated TNF, IL-8 and IL-6 levels in supernatants of normal, differentiated cells, but not in carcinoma cells treated with HAMLET (21–42 µM, 6 h). Data are means ± SEMs of triplicates from 3 independent experiments. (**E**) The innate immune response of healthy cells (IL-6, IL-8 and TNF-α) was inhibited by amiloride and TNF-α expression by BaCl_2_. qRT-PCR quantification of cytokine mRNA-levels in normal, differentiated cells exposed to HAMLET (35 µM, 1 hour).

The difference between tumor cells and healthy, differentiated cells was further examined by comparing the transcriptional responses to HAMLET (35 µM, 1 h) in A498 kidney carcinoma and healthy differentiated kidney cells. Gene expression was much less strongly affected (2064 versus 4424 genes in kidney carcinoma cells, Figure 8A, 35 µM, 1 h) and healthy cells exhibited a transient, rather than a sustained response (Figure 8A,B). No p38 MAPK- or HSP27 phosphorylation was detected (Figure S6D). In A498 kidney carcinoma cells, the p38 MAPK pathway again emerged as the top-scoring canonical pathway (Figure S5), confirming the results from lung carcinoma cells. The p38 MAPK signaling pathway genes were down-regulated in healthy, differentiated cells at early time points, with decreased expression of MKK3 and p38 MAPK after 60 and 75 min of HAMLET exposure (Figure 8C). Moreover, other death-related signaling pathways showed no significant regulation by HAMLET in normal, differentiated cells.

Pathways significantly regulated by HAMLET in normal cells were involved in innate immune regulation (IL-6 pathway, Figure 8C) and glucocorticoid signaling. Prominently upregulated genes included IL-1, IL-6, c-Jun, c-Fos, IκB and TNF-α. HAMLET also triggered IL-6, IL-8, and TNF-α secretion in healthy, differentiated cells but not in carcinoma cells (Figure 8D). The innate immune response to HAMLET in normal, differentiated cells was shown to require ion channels (Figure 8E). Amiloride abolished the increase in IL-6 and similar results were seen for IL-8 and TNF-α. BaCl_2_ reduced HAMLET- induced TNF-α expression but did not inhibit the IL-6 and IL-8 responses.

These results suggest that through differential ion channel activation, HAMLET stimulates different biological end points in tumor cells and normal, differentiated cells.

## Discussion

Ion channels are frequently dysregulated in human cancers due to gene amplifications, epigenetic regulation or splice variants of ion channel-encoding genes (for reviews, see [1], [9], [10], [11], [12]). Targeting ion fluxes should thus be a feasible strategy for anti-cancer therapy and a recent high throughput small molecule screen identified a tumoricidal potassium ionophore, particularly effective against cancer stem cells [45]. The therapeutic potential of ion channel modulators remains underexploited, however, due in part to a lack of tumor-specific channel modulators. Here, we identify a novel, non-selective cation channel in HAMLET treated tumor cells and provide evidence that the death response of tumor cells to HAMLET is caused, in part, by ion fluxes, initiating a broad and eventually lethal response, which distinguished carcinoma cells from normal, differentiated cells. This tumor-selective death through ion channel perturbation is particularly interesting, as it suggests a unifying mechanism for tumor cell death.

HAMLET perturbs the structure of biological membranes [24]. Artificial membranes composed of egg yolk or soybean lipids and lacking protein receptors responded to HAMLET with shape change, elongation and increased fluidity. The lipid or protein alone did not show these effects, suggesting that the complex has unique membrane integration properties. In addition, leakage of vesicular contents has been detected, suggesting that the membranes barrier function is altered by HAMLET [24], [46]. Mechanosensitive ion channels are membrane proteins that undergo a conformational change in response to mechanical forces that alter membrane tension voltage, pH, matrix interactions and growth factor receptor activity [47] and such channels respond to stretch along the plane of the membrane, in the absence of ‘‘specific’’ receptors [48]. Based on its direct effects on lipid bilayers, effects of HAMLET on the mechanosensing homeostasis are likely to occur. The observed inside out activation of ion fluxes by HAMLET is consistent with this hypothesis, as specific membrane orientation was not required for such fluxes to be initiated. If HAMLET itself is involved in channel formation, a direct interaction of HAMLET with the ion channel blockers may also occur.

One K^+^, Na^+^ and Ca^2+^-permeable, nonselective cation channel, was detected after HAMLET treatment. Unlike membrane pores, our whole cell patch clamp data show a marked time- and voltage-dependent inactivation. Furthermore, the permeability profile of the flux pathway is inconsistent with simple diffusion. Interestingly, the biophysical and pharmacological characteristics of this channel differ from those of classical TRP-, ENaC- or CNG channels and the activation of the channel by HAMLET in excised membrane patches appears to exclude signaling-dependent pathways such as the formation of an ATP releasing pore leading to secondary activation of ATP-sensitive ion channels, as seen for some toxins [49]. In addition, compared to the Ca^2+^ ionophore A23187, HAMLET caused a less drastic increase in [Ca^2+^]_i_ and the ionophore did not trigger a death response resembling that in HAMET treated cells, including changes in morphology, confirming that in contrast to recent suggestions [50], [51], [52], HAMLET does not merely disrupt membrane integrity by fatty acid delivery but triggers specific ion fluxes and a controlled cellular response.

This study proposes that the activation of ion fluxes in response to HAMLET may be conserved among cancer cells. Despite their different origins, lung carcinoma, kidney carcinoma, cervical carcinoma and lymphoma cells shared this response to HAMLET and pharmacologic inactivation of ion fluxes rescued these cells from HAMLET-induced death. The rapid kinetics of cell death in response to HAMLET and the insensitivity to classical cell death inhibitors [53] suggests that a new cell death modality might be activated. The findings suggest that cation fluxes coupled to early activation of the p38 MAPK signaling pathway may be involved and potential candidates activated by p38 MAP kinase include a wide range of kinases (including for example MAPKAPK2, MNK1, and MSK1) as well as transcription factors (CHOP, p53, ATF-1/2/6 and others). The effect of p38 inhibition was limited to about six hours, however, reflecting the time of action of the inhibitors or the involvement of additional, p38 independent mechanisms that later overrule this pathway. Identifying these, as yet unknown downstream effectors of the death response would be of great significance to understand how the initiation of a response at the membrane level may be transmitted into conserved cell death signals.

Apoptotic cell death is associated with a decrease in [K^+^]_i_ to 30–50 mM [54], [55], [56]. The reduction of [K^+^]_i_ is required for caspase activation in Jurkat cells [55] as well as for DNA degradation [57]. Previously, decreased [K^+^]_i_ and increased [Na^+^]_i_ were shown to correlate with caspase-3 activation in cisplatin-induced apoptosis of mammary cancer-derived cells [56]. Obviously, an increase in Ca^2+^ and a net loss of osmolytes and volume is an early and essential step in apoptotic cell death and this process involves the opening of ion channels [55], [58]. While these mechanistic links between channel opening and apoptotic cell death are established, HAMLET treated cells do not die by apoptosis. An apoptotic response is activated but cell death is caspase independent, proceeding with virtually similar kinetics in the presence of pan-caspase inhibitors or in caspase 3-deficient cells [29]. Furthermore, the characteristics of the channel activated by HAMLET have, to our knowledge, never been described in this context.

The results further indicate that differences at the level of the plasma membrane may distinguish normal differentiated cells from tumor cells. Normal cells exhibited no detectable membrane perturbations, a modest transcriptional response, mainly involving innate immunity and no effect on p38 MAPK signaling. The results also raise the question of whether the combined activation of tumor cells and normal differentiated cells might result in essential biological cooperativity. The innate immune response of normal cells to HAMLET would ideally serve to activate cells that scavenge and digest the remnants of dying cells and provide a suitable immune environment for cancer cell removal. We speculate that HAMLET’s efficiency as a selective tumoricidal agent combined with the innate immune response of normal differentiated cells may contribute to the low toxicity of HAMLET in clinical studies as well as its beneficial effects. The ion channel-dependent effector response in tumor cells accompanied by a beneficial innate immune response in surrounding tissues may serve as a two-tiered approach to killing cancer cells while maintaining tissue integrity.

## Supporting Information

Figure S1 **HAMLET does not affect cyclic nucleotide-gated (CNG) channels.** (A) Current-voltage relationship of cGMP activated currents in A549 cells. Using the ramp protocol described in Materials and Methods, currents were measured under standard conditions with an additional 1 mM 8Br-cGMP in the pipette solution. Representative of 2 independent experiments (B) Time dependent characteristics of the cGMP activated currents. In the same cell as in A, time dependent characteristics were investigated using a 1 s protocol stepping from −100 mV to +100 mV in 20 mV increments. Representative of two independent experiments. (C–D) The CNG channel inhibitor L-cis-diltiazem (LCD) does not inhibit HAMLET stimulated Ca^2+^ signaling. [Ca^2+^]_i_ was measured using the Ca^2+^ sensitive fluorophore, Fura-2 (Molecular Probes). Cells grown on size #1 coverslips overnight were loaded 30 min with 4 µM Fura-2 in normal growth medium before being installed in a microscope perfusion chamber with constant perfusion with Krebs solution (in mM: 150 NaCl, 6 KCl, 1 MgCl_2_, 1.5 CaCl_2_, 10 HEPES, 10 Glucose, pH 7.4 using NaOH). Fura-2 fluorescence of individual cells was measured through a through a 40×/1.4 NA oil immersion objective (Olympus, Tokyo, Japan) using an Imic2000 microscope with a PolychromeV monochromator as the light source (Till Photonics, Gräfelfing, Germany), a Chroma 79001ET filterset (Chroma Technology, Bellows Falls, VT, USA ), and digitized by an Ixon 885 camera (Andor, Belfast, N. Ireland). Signals between 470–550 nm following 20 ms excitation at 340 nm or 380 nm, were measured in 1 s intervals. Microscope control, signal visualization and analysis were performed in Live Acquisition software (Till Photonics). The presence of HAMLET (35 µM) or LCD (200 µM) in the superfusate is indicated by the top bar. Each trace indicates the Fura-2 ratio of an individual cell. Representative of 2 independent experiments. (E) A549 lung carcinoma cells were pretreated with L-cis-diltiazem (LCD) and HAMLET-treated as shown. There was no significant inhibitory effect of LCD on cell death.
(TIF)

Figure S2 **Amiloride and BaCl_2_ rescue HeLa cells from HAMLET-induced cell death.** (A) Viability of HeLa cells after exposure to HAMLET (21, 28 or 35 µM, 3 h), quantified by ATP levels or Trypan blue exclusion. BaCl_2_ inhibited cell death but GdCl_3_, Ruthenium Red had no effect (B) Amiloride inhibited the tumoricidal effect of HAMLET but tetranidrine showed no effect.
(TIF)

Figure S3 **Amiloride and BaCl_2_ rescue Jurkat cells from HAMLET-induced cell death.** Jurkat lymphoma cells were pre-incubated with ion channel inhibitors as indicated and treated with HAMLET (7–21 µM, 3 hours). Cell death was quantified by trypan blue exclusion or ATP levels. (A) Amiloride or BaCl_2_ pretreated cells were rescued but GdCl_2_ had no effect (B) Ruthenium Red or tetrandrine did not rescue the cells from HAMLET –induced death. (C) Prolonged rescue (24 hours) by amiloride and BaCl_2_ of A549 lung carcinoma cells treated with HAMLET. (D) A combination of Amiloride and BaCl_2_ completely rescued tumor cells from the lethal effects of HAMLET. Removal of extra-cellular calcium did not reduce cell death. (E) Neither inhibition of ER Ca^2+^ release by U73122, nor depletion of extracellular Ca^2+^ by EDTA rescued the cells from HAMLET-induced death.
(TIF)

Figure S4 **Effect of ion channel inhibitors on HAMLET uptake by lung carcinoma cells.** Internalization of Alexa-568 fluor labeled HAMLET by tumor cells (35 µM, 1 hour, visualized by epifluorescence microscopy. Amiloride or BaCl_2_ inhibited internalization, leaving HAMLET associated with the cell surface. WGA scale bar = 100 µm.
(TIF)

Figure S5 **Differential expression of genes in the p38 MAPK-signaling pathway.** A498 human kidney carcinoma cells were exposed to HAMLET for three hours and differentially expressed genes were functionally categorized using Ingenuity Pathway Analysis. The p38-signaling pathway was identified as the top-scoring pathway.
(TIF)

Figure S6 **MAPK phosphorylation in response to HAMLET.** (A) Lung carcinoma cells downregulate ERK1/2 and activate p38α activity in response to HAMLET. (B) Kidney carcinoma cells respond to HAMLET by phosphorylating p38α, p38β and p38γ as well as the downstream target HSP27, while ERK1/2 was dephosphorylated. Lysates of kidney carcinoma cells (A498) exposed to HAMLET (35 µM) for 30 minutes. Membranes with phospho-specific antibodies were probed with protein lysates from HAMLET- or PBS-treated (control) carcinoma cells. Protein phosphorylation was quantified using ImageJ. Data are means ± SDs. (C) p38 inhibition by SB202190 abrogates phosphorylation of p38 and HSP27. Lung carcinoma cells were preincubated with SB202190 (20 µM, 30 minutes) and HAMLET-treated (35 µM, 30 minutes). (D) Normal, differentiated cells do not activate p38 in response to HAMLET. Pediatric kidney cells in primary culture were treated with HAMLET (49 µM, 30 minutes). (E) p38 inhibition (BIRB796, 10 µM) rescued carcinoma (A549 and A498) cells from death in response to HAMLET (7–35 µM, 3 h). Viability was quantified as ATP levels. (F) BIRB796 (10 µM) diminishes the morphological changes associated with HAMLET-induced cell death (35 µM, 3 h).
(TIF)
