# Supplementary figures and images for: A Unifying Mechanism for Cancer Cell Death through Ion Channel Activation by HAMLET
Source: PLoS One. 2013 Mar 7;8(3):e58578. doi: 10.1371/journal.pone.0058578 (PMC3591364; doi:10.1371/journal.pone.0058578)

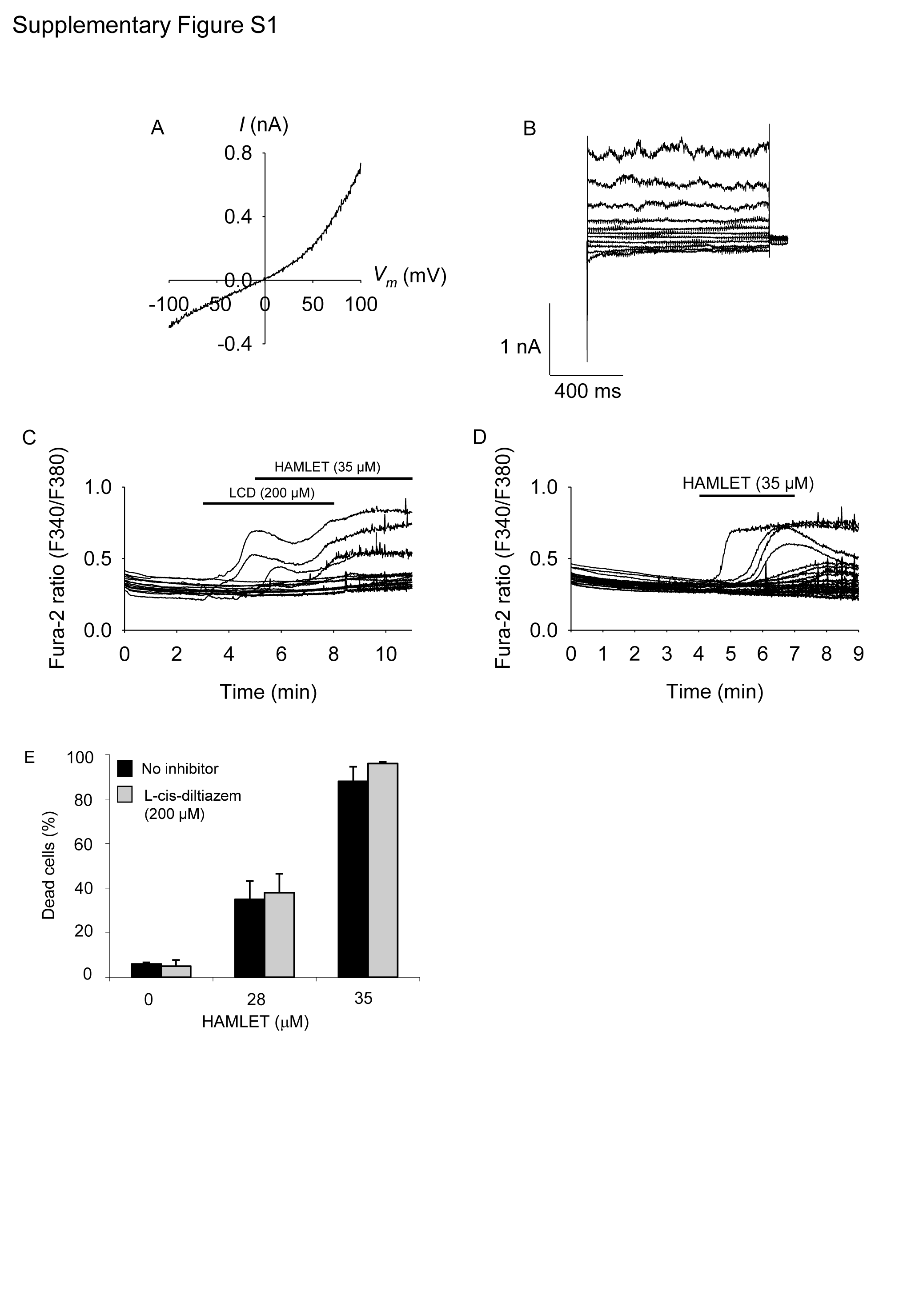

Supplement: Figure S1 — HAMLET does not affect cyclic nucleotide-gated (CNG) channels. (A) Current-voltage relationship of cGMP activated currents in A549 cells. Using the ramp protocol described in Materials and Methods, currents were measured under standard conditions with an additional 1 mM 8Br-cGMP in the pipette solution. Representative of 2 independent experiments (B) Time dependent characteristics of the cGMP activated currents. In the same cell as in A, time dependent characteristics were investigated using a 1 s protocol stepping from −100 mV to +100 mV in 20 mV increments. Representative of two independent experiments. (C–D) The CNG channel inhibitor L-cis-diltiazem (LCD) does not inhibit HAMLET stimulated Ca2+ signaling. [Ca2+]i was measured using the Ca2+ sensitive fluorophore, Fura-2 (Molecular Probes). Cells grown on size #1 coverslips overnight were loaded 30 min with 4 µM Fura-2 in normal growth medium before being installed in a microscope perfusion chamber with constant perfusion with Krebs solution (in mM: 150 NaCl, 6 KCl, 1 MgCl2, 1.5 CaCl2, 10 HEPES, 10 Glucose, pH 7.4 using NaOH). Fura-2 fluorescence of individual cells was measured through a through a 40×/1.4 NA oil immersion objective (Olympus, Tokyo, Japan) using an Imic2000 microscope with a PolychromeV monochromator as the light source (Till Photonics, Gräfelfing, Germany), a Chroma 79001ET filterset (Chroma Technology, Bellows Falls, VT, USA ), and digitized by an Ixon 885 camera (Andor, Belfast, N. Ireland). Signals between 470–550 nm following 20 ms excitation at 340 nm or 380 nm, were measured in 1 s intervals. Microscope control, signal visualization and analysis were performed in Live Acquisition software (Till Photonics). The presence of HAMLET (35 µM) or LCD (200 µM) in the superfusate is indicated by the top bar. Each trace indicates the Fura-2 ratio of an individual cell. Representative of 2 independent experiments. (E) A549 lung carcinoma cells were pretreated with L-cis-diltiazem (LCD) and HAMLET-trea [file pone.0058578.s001.tif]

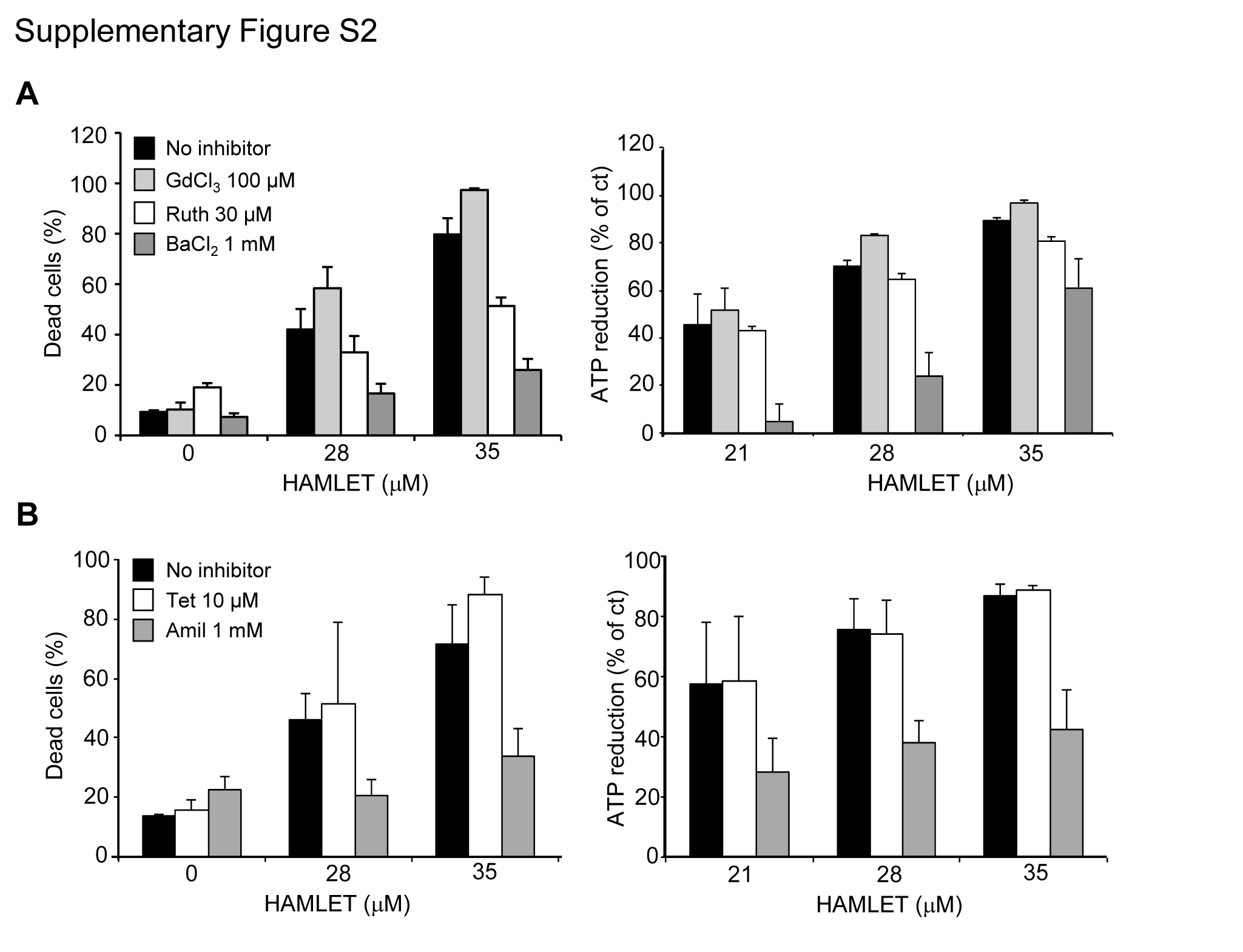

Supplement: Figure S2 — Amiloride and BaCl2 rescue HeLa cells from HAMLET-induced cell death. (A) Viability of HeLa cells after exposure to HAMLET (21, 28 or 35 µM, 3 h), quantified by ATP levels or Trypan blue exclusion. BaCl2 inhibited cell death but GdCl3, Ruthenium Red had no effect (B) Amiloride inhibited the tumoricidal effect of HAMLET but tetranidrine showed no effect. (TIF) [file pone.0058578.s002.tif]

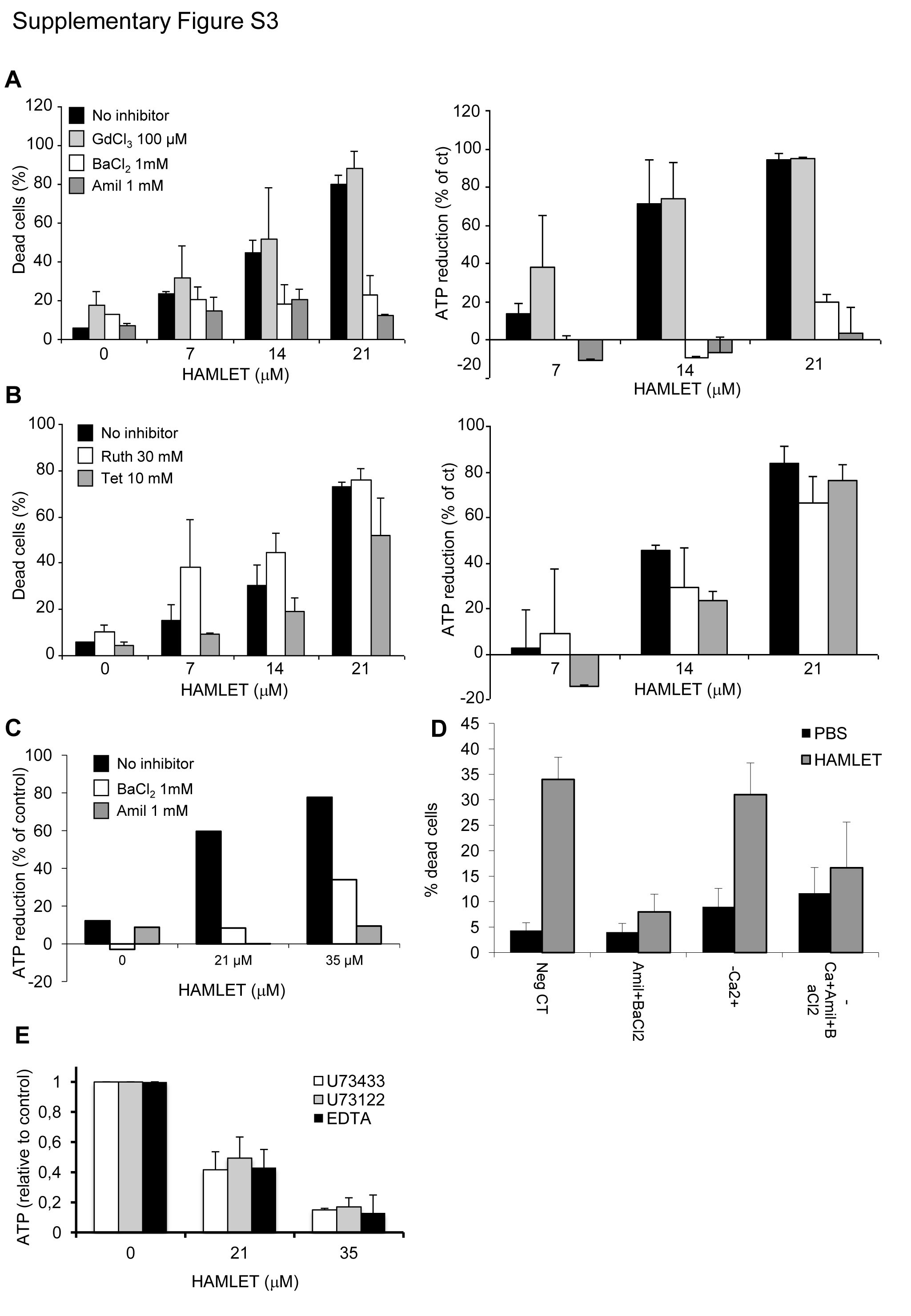

Supplement: Figure S3 — Amiloride and BaCl2 rescue Jurkat cells from HAMLET-induced cell death. Jurkat lymphoma cells were pre-incubated with ion channel inhibitors as indicated and treated with HAMLET (7–21 µM, 3 hours). Cell death was quantified by trypan blue exclusion or ATP levels. (A) Amiloride or BaCl2 pretreated cells were rescued but GdCl2 had no effect (B) Ruthenium Red or tetrandrine did not rescue the cells from HAMLET –induced death. (C) Prolonged rescue (24 hours) by amiloride and BaCl2 of A549 lung carcinoma cells treated with HAMLET. (D) A combination of Amiloride and BaCl2 completely rescued tumor cells from the lethal effects of HAMLET. Removal of extra-cellular calcium did not reduce cell death. (E) Neither inhibition of ER Ca2+ release by U73122, nor depletion of extracellular Ca2+ by EDTA rescued the cells from HAMLET-induced death. (TIF) [file pone.0058578.s003.tif]

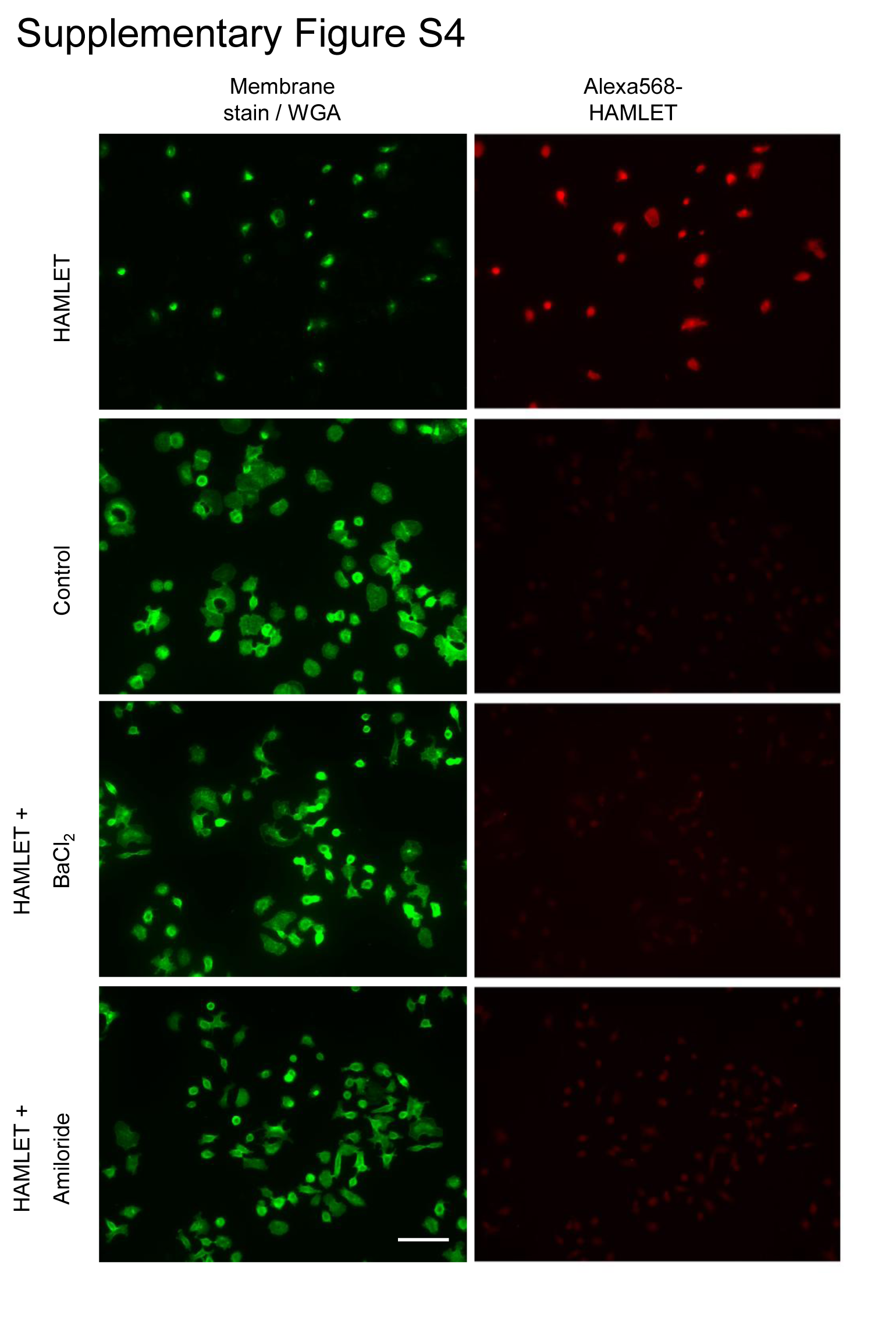

Supplement: Figure S4 — Effect of ion channel inhibitors on HAMLET uptake by lung carcinoma cells. Internalization of Alexa-568 fluor labeled HAMLET by tumor cells (35 µM, 1 hour, visualized by epifluorescence microscopy. Amiloride or BaCl2 inhibited internalization, leaving HAMLET associated with the cell surface. WGA scale bar = 100 µm. (TIF) [file pone.0058578.s004.tif]

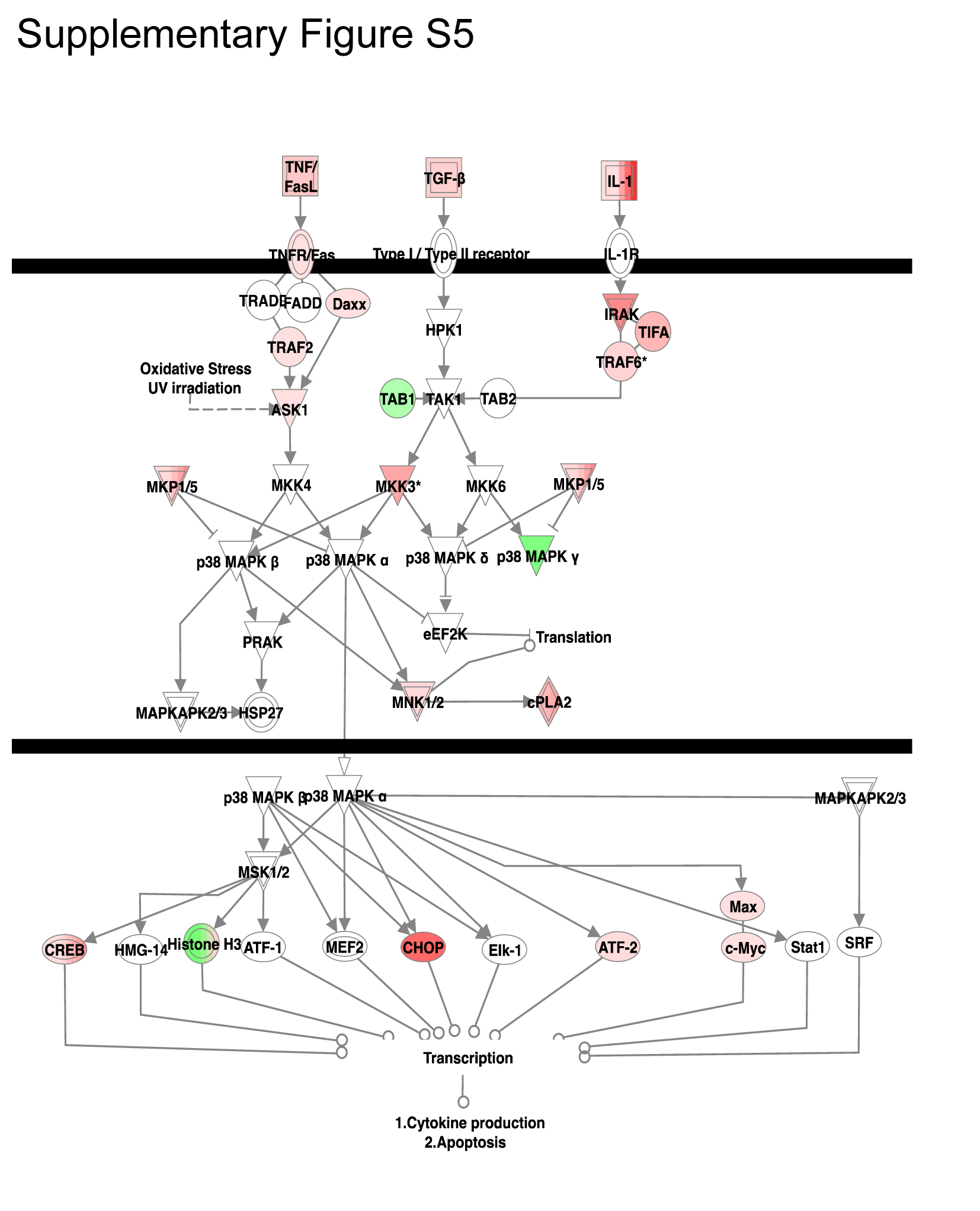

Supplement: Figure S5 — Differential expression of genes in the p38 MAPK-signaling pathway. A498 human kidney carcinoma cells were exposed to HAMLET for three hours and differentially expressed genes were functionally categorized using Ingenuity Pathway Analysis. The p38-signaling pathway was identified as the top-scoring pathway. (TIF) [file pone.0058578.s005.tif]

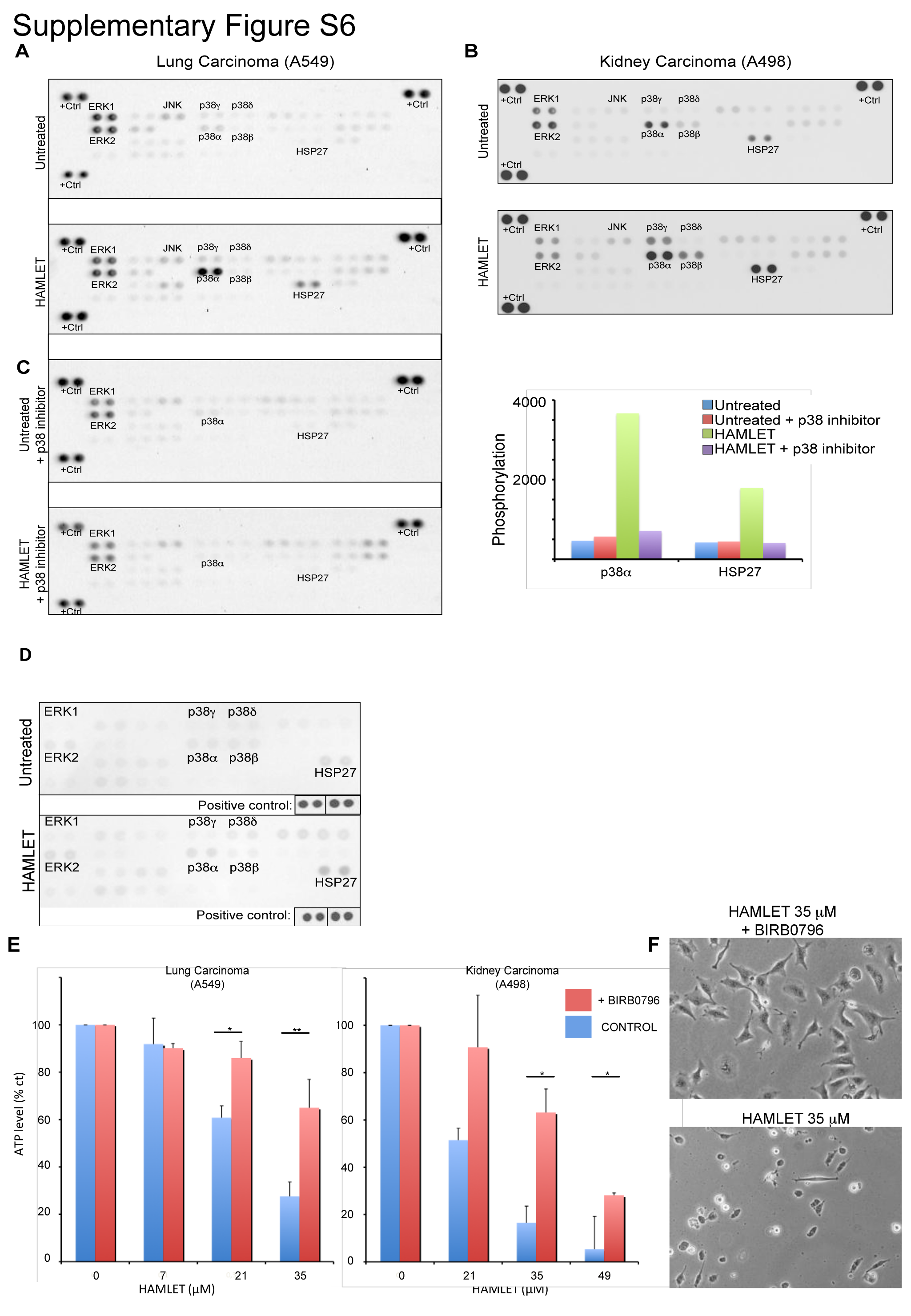

Supplement: Figure S6 — MAPK phosphorylation in response to HAMLET. (A) Lung carcinoma cells downregulate ERK1/2 and activate p38α activity in response to HAMLET. (B) Kidney carcinoma cells respond to HAMLET by phosphorylating p38α, p38β and p38γ as well as the downstream target HSP27, while ERK1/2 was dephosphorylated. Lysates of kidney carcinoma cells (A498) exposed to HAMLET (35 µM) for 30 minutes. Membranes with phospho-specific antibodies were probed with protein lysates from HAMLET- or PBS-treated (control) carcinoma cells. Protein phosphorylation was quantified using ImageJ. Data are means ± SDs. (C) p38 inhibition by SB202190 abrogates phosphorylation of p38 and HSP27. Lung carcinoma cells were preincubated with SB202190 (20 µM, 30 minutes) and HAMLET-treated (35 µM, 30 minutes). (D) Normal, differentiated cells do not activate p38 in response to HAMLET. Pediatric kidney cells in primary culture were treated with HAMLET (49 µM, 30 minutes). (E) p38 inhibition (BIRB796, 10 µM) rescued carcinoma (A549 and A498) cells from death in response to HAMLET (7–35 µM, 3 h). Viability was quantified as ATP levels. (F) BIRB796 (10 µM) diminishes the morphological changes associated with HAMLET-induced cell death (35 µM, 3 h). (TIF) [file pone.0058578.s006.tif]
